# Asymmetric dimethylarginine (ADMA) accelerates renal cell fibrosis under high glucose condition through NOX4/ROS/ERK signaling pathway

**DOI:** 10.1038/s41598-020-72943-2

**Published:** 2020-09-29

**Authors:** Isaivani Jayachandran, Saravanakumar Sundararajan, Saravanakumar Venkatesan, Sairaj Paadukaana, Muthuswamy Balasubramanyam, Viswanathan Mohan, Nagaraj Manickam

**Affiliations:** Department of Vascular Biology, Madras Diabetes Research Foundation & Dr. Mohan’s Diabetes Specialities Centre, WHO Collaborating Centre for Non-Communicable Diseases Prevention and Control & ICMR Center for Advanced Research On Diabetes, Chennai, India

**Keywords:** Biochemistry, Cell biology, Cell signalling, Kidney diseases

## Abstract

We previously reported that the circulatory level of Asymmetric dimethylarginine (ADMA), an endogenous competitive inhibitor of nitric oxide synthase, was increased in diabetic kidney disease patients. However, the mechanism and the role of ADMA in diabetic kidney injury remain unclear. Hence, our principal aim is to investigate the causal role of ADMA in the progression of renal cell fibrosis under high glucose (HG) treatment and to delineate its signaling alterations in kidney cell injury. High Glucose/ADMA significantly increased fibrotic events including cell migration, invasion and proliferation along with fibrotic markers in the renal cells; whereas ADMA inhibition reversed the renal cell fibrosis. To delineate the central role of ADMA induced fibrotic signaling pathway and its downstream signaling, we analysed the expression levels of fibrotic markers, NOX4, ROS and ERK activity by using specific inhibitors and genetic manipulation techniques. ADMA stimulated the ROS generation along with a significant increase in NOX4 and ERK activity. Further, we observed that ADMA activated NOX-4 and ERK are involved in the extracellular matrix proteins accumulation. Also, we observed that ADMA induced ERK1/2 phosphorylation was decreased after NOX4 silencing. Our study mechanistically demonstrates that ADMA is involved in the progression of kidney cell injury under high glucose condition by targeting coordinated complex mechanisms involving the NOX4- ROS-ERK pathway.

## Introduction

Diabetic kidney disease plays a major role in intensifying the mortality rate of the diabetes population globally^1^. Development of diabetic kidney disease involves several steps; one of the early steps involved in causing the diabetic kidney injury is vasculo-endothelial dysfunction^2^. It is known that nitric oxide (NO) plays a major role in maintaining vasculature and a reduction in the bioavailability of NO leads to vascular dysfunction^3^. Increase in the level of asymmetric dimethylarginine (ADMA), an endogenous inhibitor of nitric oxide synthase (NOS) results in decrease in the synthesis of nitric oxide. ADMA is synthesized by the enzyme protein methyltransferase (PRMT) from the methylated arginine protein residues during the post translational modification and metabolized subsequently by the enzyme dimethylarginine dimethylaminohydrolase (DDAH). Over the past decade there has been increasing reports suggests that under various pathophysiological pathways, ADMA plays a deleterious role. ADMA-related profibrotic alterations appear to be common in several organs, particularly in kidney, heart and liver which are more susceptible to ADMA pathogenesis. It has been known from a mice model study that DDAH1 deficiency induces the epithelial to mesenchymal transition in renal proximal tubular epithelial cells and exacerbates kidney damage^4^. Another recent study also demonstrated that DDAH alleviates myocardial fibrosis in diabetic cardiomyopathy through activation of the DDAH/ADMA/NOS/NO pathway in rats^5^. Thus, it appears that pathogenic effects of ADMA is linked to various vascular complications of diabetes and warrants appropriate therapeutic strategies to counter act.

Amino acids are translocated across the cells via different transporters. l-arginine and methylarginines are transported via cationic amino acid transporters (CAT)^6^. It is believed that CAT is the main transporter of ADMA in the kidney fibroblast. It has recently been found that mitochondrial carrier SLC25A2 is also involved in the transport of ADMA^7^. We assume that under high glucose condition, CAT could act as a main contributor in the fibroblast for transporting ADMA in and out of the cells.

In our previous report, we have shown that under high glucose condition, the ADMA metabolizing enzyme DDAH 1 activity was significantly reduced resulting in the increase in the intracellular concentration of ADMA^8^. Further, we have shown that ADMA levels are increased in type 2 patients with albuminuria and it can be used as a prognostic biomarker for diabetic kidney disease^8^.

ECM turnover is vital for maintaining the normal structure and function of the kidney. We hypothesize that reduction in nitric oxide level by ADMA will affect renal vasculature and alters ECM turnover. Accumulation of ECM proteins in the renal mesangium resulting in interstitial fibrosis is believed to be an important factor for the development of vascular dysfunction wherein the epithelial cells to mesenchymal transition occurs. Increasing evidence suggests that the transition of epithelial to mesenchymal cells play a vital role in the progression of kidney fibrosis^9^. During this process, the cells are directed to various pathways leading to the formation of fibrotic tissue expressing increased extracellular matrix proteins such as fibronectin (FN), and alpha-smooth muscle actin (α-SMA), collagen, among others. These pathological changes are believed to lead chronic kidney disease (CKD) and result in End Stage Renal Disease (ESRD) in diabetes patients. In kidney, under pathological conditions such as diabetes, cells are involved in the over-production of extracellular matrix proteins, in which activated fibroblasts and mesangial cells play a key role^10^.

Further, oxidative stress has a noxious role in the progression of diabetic kidney disease by playing a common connector role between the major pathophysiological pathways. Oxidative stress is induced via a vast variety of sources, of these the primary source and most pre-dominant isoform in the kidney cells are NAD(P)H oxidase 4 (NOX-4). Renal cells mainly fibroblasts, mesangial cells and proximal tubular cells expresses NOX4^11^. Under high glucose milieu, imbalance in the intracellular glucose homeostasis increases the ROS production leading to the activation of several pathophysiological pathways^12,13^. However the role of ADMA in the ROS generation and downstream signaling in fibrosis is not known. Thus, comprehensive molecular studies of ADMA induced renal fibrosis are essential as they could identify novel drug targets and also could facilitate the early intervention of diabetic kidney disease (DKD).

Hence, in this study we aimed to delineate the molecular mechanism of ADMA in causing the kidney injury under high glucose milieu (invitro) in the rat fibroblasts using Normal rat kidney cells (NRK-49F) and Rat mesangial cells (RMC).

## Methods

### Cell culture

Normal rat kidney fibroblasts and Rat Mesangial cells was obtained from the American Type Cell Collection (ATCC, Manassas, VA, USA). Cells were cultured in Dulbecco’s Modified Eagle Medium (DMEM; Gibco, Carlsbad, CA) supplemented with 10% foetal bovine serum (FBS; Invitrogen, Carlsbad, CA, USA) and 1% penicillin/streptomycin (Sigma, St. Louis, MO, USA) in a CO_2_ incubator with a humidified atmosphere of 5% CO_2_ at 37 °C.

Cells were seeded in 12-well plate and 6-well plate for gene expression and protein expression studies, respectively. Once cells reached 60–70% confluence, they were serum starved for 24 h and treated with respective conditions. Cells under different treatment conditions were washed with 1X PBS and harvested using TRIzol for gene expression studies and RIPA cell lysis buffer for protein expression studies. In our pilot studies, we used mannitol for analyzing osmotic pressure normalization. We found that mannitol did not cause any effect in the analyzed protein expression patterns in both the NRK49-F and RMC cell lines. Therefore, we continued our subsequent studies without using mannitol.

### Reagents

*N*G, *NG*-Dimethylarginine dihydrochloride (ADMA), l-arginine, Diphenyleneiodonium (DPI) and GW4064 were purchased from Sigma-Aldrich (St. Louis, MO). 1,4-diamino-2,3-dicyano-1,4-bis[2- aminophenylthio] butadiene (UO126) was purchased from Cell Signaling Technology (Beverly, MA, USA).

### Cell migration assay

A wound was created by scratching the monolayer of cells with a pipette tip and the scratches were photographed with inverted phase contrast microscope. After a wash with 1 × PBS, each well were replaced with fresh medium and pre-treated with GW4064/ARG for 30 min and then treated with or without ADMA/HG. (GW4064, which is a classical FXR agonist has also been used as an agonist for DDAH1. DDAH gene sequencing revealed the presence of FXR response element 90 kb downstream of transcription initiation site & functional analysis showed the transcriptional activation of FXR response element on treatment with GW4064^14^). After 16 h of incubation, a second set of photograph of the scratches were taken. The migration was determined by analysing the images and represented as the increase in the percentage of closed area.

### Transwell invasion assay

Cell invasion was measured using transwell chamber (Corning Costar Corp., Cambridge, MA, USA). The top chamber was coated with 0.2% gelatin and incubated for overnight at 37 °C in the incubator and the next day, serum starved cells were seeded in top chamber. The lower chambers were filled with medium pre-treated with inhibitors of ADMA such as GW4064/ARG in the presence or absence of agonists. After 24 h incubation, the number of migrated cells in the insert was counted from three different power fields to quantify the number of cells invaded under different conditions.

### Immunocytochemistry

NRK-49F cells were cultured in 35 mm dishes containing cover slips and serum deprived for 16 h. Cells were then treated with appropriate agonists and inhibitors. After the treatment period, cells were fixed with 3.7% paraformaldehyde on ice for 10 min, washed and then permeabilized with 0.1% Triton X-100 in PBS for 5 min. Cells were washed again and then blocked with 1% BSA for 1 h at room temperature. Cells were then incubated with primary antibody in PBS for overnight. After three washes, cells were incubated with 1:1000 diluted secondary antibody labelled with Alexa Fluor (Anti-mouse@649, Bio‐Rad, Hercules, CA, Anti-rabbit@488, Abcam, Inc., Cambridge, MA) for 3 h at 4 °C. The coverslips were mounted on slides with the mounting media having DAPI. The cells were then visualized and imaged under confocal microscopy (Carl Zeiss, Oberkochen, Germany)^15^.

### BrdU cell proliferation assay

Cells were appropriately conditioned with and without inhibitor/activator and the cell proliferation was detected using a BrdU proliferation kit (BioVision, Inc. Milpitas, CA, USA) in accordance with the manufacturer's instructions.

### Transfection of siRNA

siRNA oligonucleotides targeted specifically to rat DDAH 1 and NOX-4 (Qiagen, Hilden, Germany) were used in this experiment. Cells were seeded on 6-well plate in serum free media for overnight. HiPerfect transfection reagent (Qiagen, Hilden, Germany) were used to transfect the cells with 20 nM of respective siRNAs as per the manufacturer’s instructions. Scrambled siRNA (20 nM) were used as transfection control. After the respective treatments, cells were harvested using RIPA cell lysis buffer for studying protein expressions.

### Immunoblotting

Cells were lysed using RIPA buffer and the protein content was quantified by using Bradford’s reagent (Bio‐Rad, Hercules, CA) as per manufacturer’s instructions. Equal quantity of protein per lane was separated by SDS-PAGE. The separated proteins were then transferred onto the PVDF membrane. The membranes were blocked for 2 h with 5% blocking buffer (5% non-fat dry milk powder in 1X PBST). After washing in 1X PBST, membranes were incubated in respective primary antibodies, Fibronectin, α-SMA, NOX-4, AGXT2 (Novus Biologicals, Littleton, CO), p-eNOS, p-ERK, ERK, (Cell Signaling Technology (Beverly, MA, USA)), DDAH1 and GAPDH, (Santa Cruz Biotechnology, Santa Cruz, CA, USA), eNOS (Abcam, Cambridge, UK ),overnight at 4 °C and following the secondary antibody for 3 h. Protein bands were visualized using enhanced chemiluminescent reagent (Bio‐Rad, Hercules, CA) using Bio-Rad gel doc instrument. Images of the band intensity were quantified using the ImageJ software (NIH, Bethesda, MD).

### Real time qPCR

The total RNA was isolated from NRK-49F cells using TRIzol (Sigma-Aldrich, St. Louis, MO) reagent as described elsewhere. 250 ng of RNA was converted to cDNA using reverse transcriptase. cDNA (50 ng) was used for real time PCR analysis of target gene of PRMT1 (Forward Primer: CTACTTTGACTCCTATGCCCAC, Reverse Primer: CTTTGAAGAGATGCCGATTGTG) using SYBR green (Takara, Kusatsu, Japan) with appropriate cycle conditions in LC96 autoanalyzer (Roche, Basel, Switzerland). ΔΔct method was used to calculate the fold stimulation by normalizing to endogenous 18S ribosomal RNA gene.

### Intracellular ROS assay

Cells were grown in 96 well plate and serum deprived for 16 h. Cells were then incubated with ADMA in a time dependent manner. Cells were then washed with PBS and loaded with 10 μM of 2′,7′-dichlorodihydrofluorescin diacetate (DCFDA) ((Sigma, St. Louis, MO) dissolved in Hank’s balanced salt solution for 30 min at 37 °C. After washing with HBSS, the levels of intracellular ROS was measured as DCF fluorescence at excitation and emission wavelengths of 488 and 520 nm, respectively in a Multimode plate reader (PerkinElmer, Waltham, MA).

### ROS assay-confocal microscopy

Cells were plated into 24 well plates containing cover slips and serum deprived for 16 h. Cells were then pre incubated for 1 h with l-arginine or GW4064 followed by addition of HG or ADMA for 24 h. Cells were then washed with Krebs–Ringer’s solution and loaded with 10 μM of DCFDA, dissolved in Krebs–Ringer’s solution for 30 min at 37 °C. Cells were washed in Krebs–Ringer’s solution and the cover slips were mounted on a glass slide and then the accumulation of ROS was visualized under confocal microscope (Carl Zeiss, Oberkochen, Germany).

### Statistical analysis

The statistical analysis was performed using GraphPad Prism software (GraphPad Prism Software, USA). All the experiments were performed for a minimum of three independent experiments. Results are expressed as the Mean ± SEM. Statistical comparisons between groups were performed by one way analysis of variance, and post hoc analysis was done using Tukey multiple comparison test using Graphpad Prism 5 software. Any difference with *p* < 0.05 was considered statistically significant.

## Results

### ADMA and high glucose triggers fibrotic events in the kidney cells

**Figure 1:**
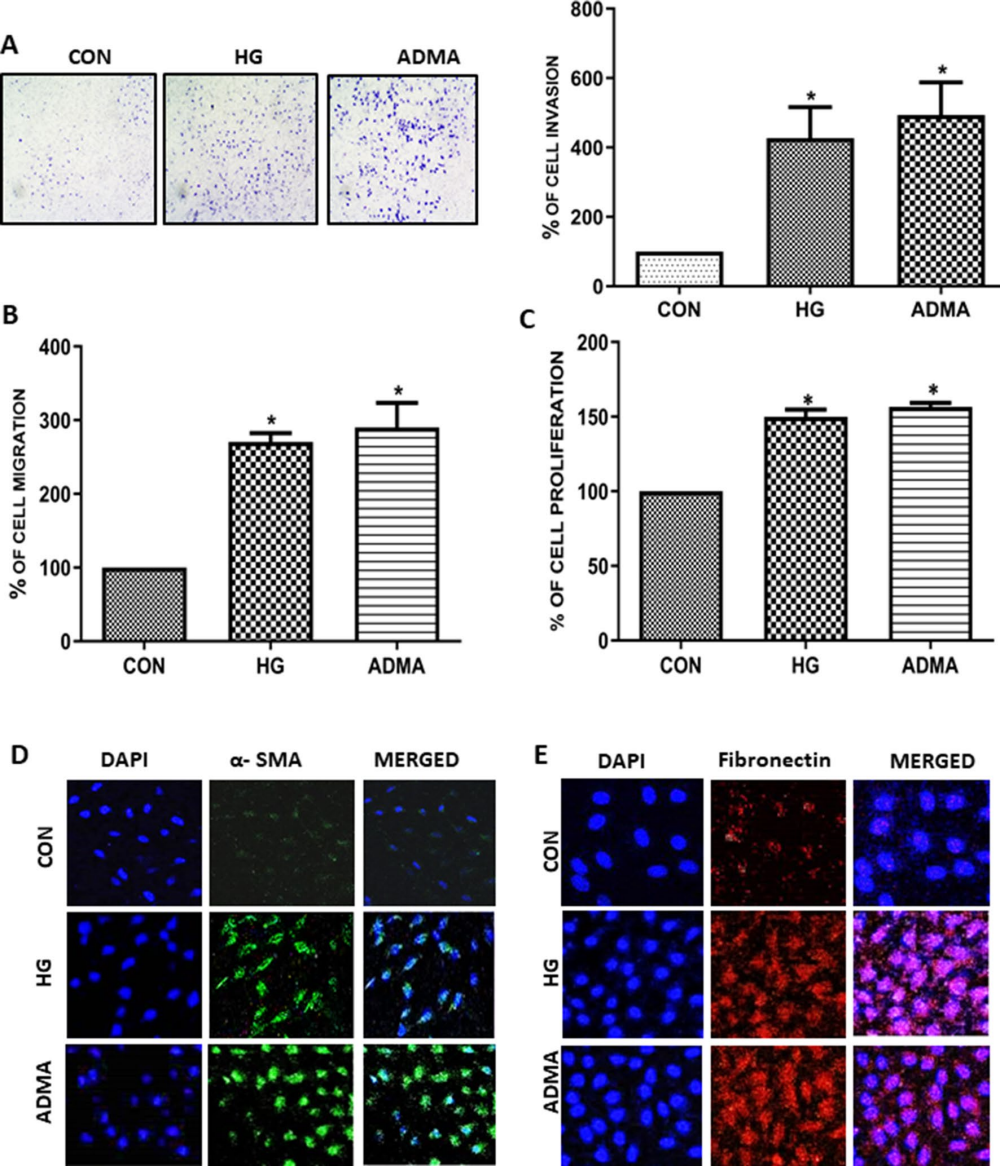
ADMA and high glucose triggers fibrotic events in the kidney cells: (**A**) Representative images of NRK-49F cells treated with HG/ADMA for 10 h in transwell and the pictures were taken in random high power fields. Cells were counted from four random microscopic fields per insert in triplicate. The migrated cell numbers were normalized to that of the control group. The results were expressed as % of invasion. (**B**) NRK-49F monolayers were scratched by using pipette tip and treated with HG/ADMA for 12 h, the percentage of wound width was calculated and the results were expressed as % of migration of HG/ADMA treated cells compared to control. (**C**) Cell proliferation was determined by BrdU incorporation assay, and the results were expressed as % of proliferation of HG/ADMA treated cells compared with control. Results are presented as Mean ± SD of three independent experiments. (**D**,**E)** Representative photomicrographs of immunofluorescence staining of α-SMA and fibronectin. *p < 0.05 compared to the Control.

Cell migration, proliferation and excessive production of matrix proteins, are the hall marks of renal cell fibrosis. To elucidate the role of ADMA in the renal cell fibrosis, we performed invasion assay, cell migration assay and proliferation assay. ADMA as well as HG significantly increased the cell migration, invasion and proliferation (Fig. 1A–C). To further elucidate and confirm the role of ADMA in fibrosis, the expression levels of differentiated fibrotic markers such as fibronectin and α-SMA were analysed as a paradigm. Interestingly, we found that both HG and ADMA significantly increased the accumulation of extra cellular matrix proteins as observed from the confocal microscopy (Fig. 1D,E) which confirms that ADMA by itself can induce myofibroblast activation/fibrotic events.

To study the effect of HG on the ADMA synthesizing enzyme, we analyzed the mRNA expression profile of PRMT1 under high glucose condition in the renal cells. Exposure to high glucose significantly upregulated the mRNA expression profile of PRMT1 in time dependent manner (Supplementary Fig. S1). Further, we have studied the effect of high glucose on ADMA metabolizing enzyme, AGXT2 an alternative metabolic enzyme involved in the catabolism of ADMA. We used dose kinetic study to analyse the expression level of AGXT2 with glucose treatment. We have found that high glucose significantly decreased the AGXT2 expression in dose dependent manner (Supplementary Fig. S2). These results substantiate our hypothesis that under high glucose condition, changes in the expression profiles of PRMT1 and AGXT2 leads to the accumulation of ADMA which in turn causes the renal cell injury.

Also, it has been well elucidated that nitric oxide is a major player in slowing the progression of renal fibrosis. We have analyzed eNOS activity under ADMA treatment. We have found that ADMA treatment significantly decreased the expression levels of phospho eNOS (Supplementary Fig. S3). Hence, we are suggesting that ADMA induced pro-fibrotic process is dependent on NOS activity.

### Role of ADMA under the high glucose milieu is critical for renal cell fibrosis

**Figure 2:**
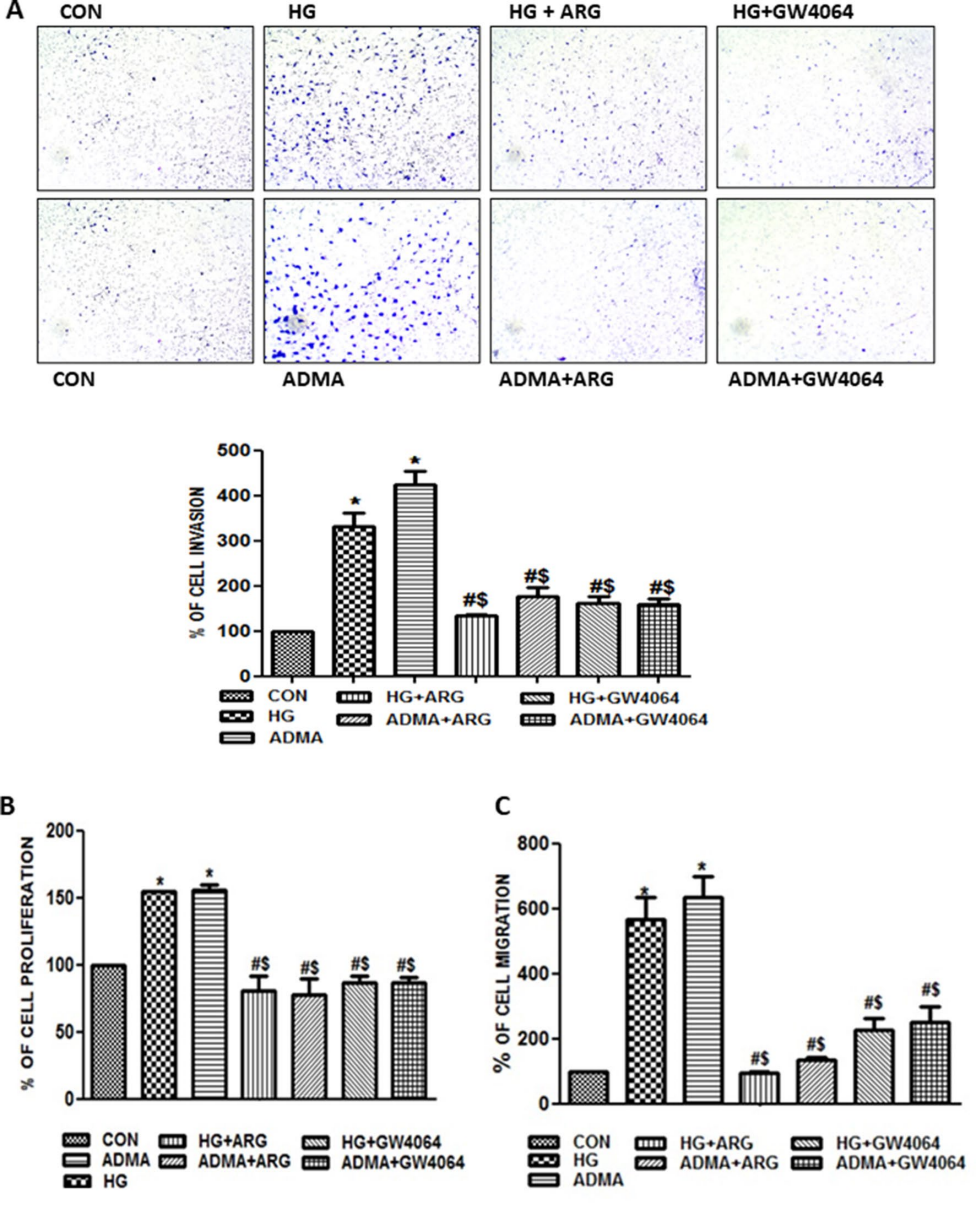
Role of ADMA under the high glucose milieu is critical for renal cell fibrosis: (**A**) Representative images of NRK-49F cells pre-treated with ARG/GW4064 for 30 min and then stimulated with HG/ADMA for 10 h in transwell and the pictures were taken in four random high power fields. Cells were counted from four random microscopic fields per insert in triplicate. The migrated cell numbers were normalized to that of the control group. The results were expressed as % of invasion. (**B**) Cell proliferation was determined by BrdU incorporation assay and the results were expressed as % of proliferation compared to control. (**C**) NRK-49F monolayers pre-treated with ARG/GW4064 for 30 min were scratched by using pipette and treated with HG/ADMA for 12 h, the percentage of wound width was calculated and the results were expressed as % of migration compared to control. Results are presented as Mean ± SD of three independent experiments. *p < 0.05 compared to the Control, ^#^p < 0.05 compared to the HG, ^$^p < 0.05 compared to the ADMA.

From the above results, we confirmed the involvement of ADMA in the fibrotic events. In order to further study the exact role of ADMA in the kidney cell injury under high glucose condition, we inhibited ADMA accumulation/activity by using DDAH 1 agonist (GW4064) and L-arginine (competitive inhibitor of eNOS). Pre-treatment with the l-Arginine or GW4064 cultured with HG (25 mM)/ADMA (100 μM) hindered ADMA induced migration (Fig. 2C), invasion (Fig. 2A) and proliferation (Fig. 2B) on the cells. This showed the central role of ADMA in the progression of kidney cell injury under high glucose condition.

**Figure 3:**
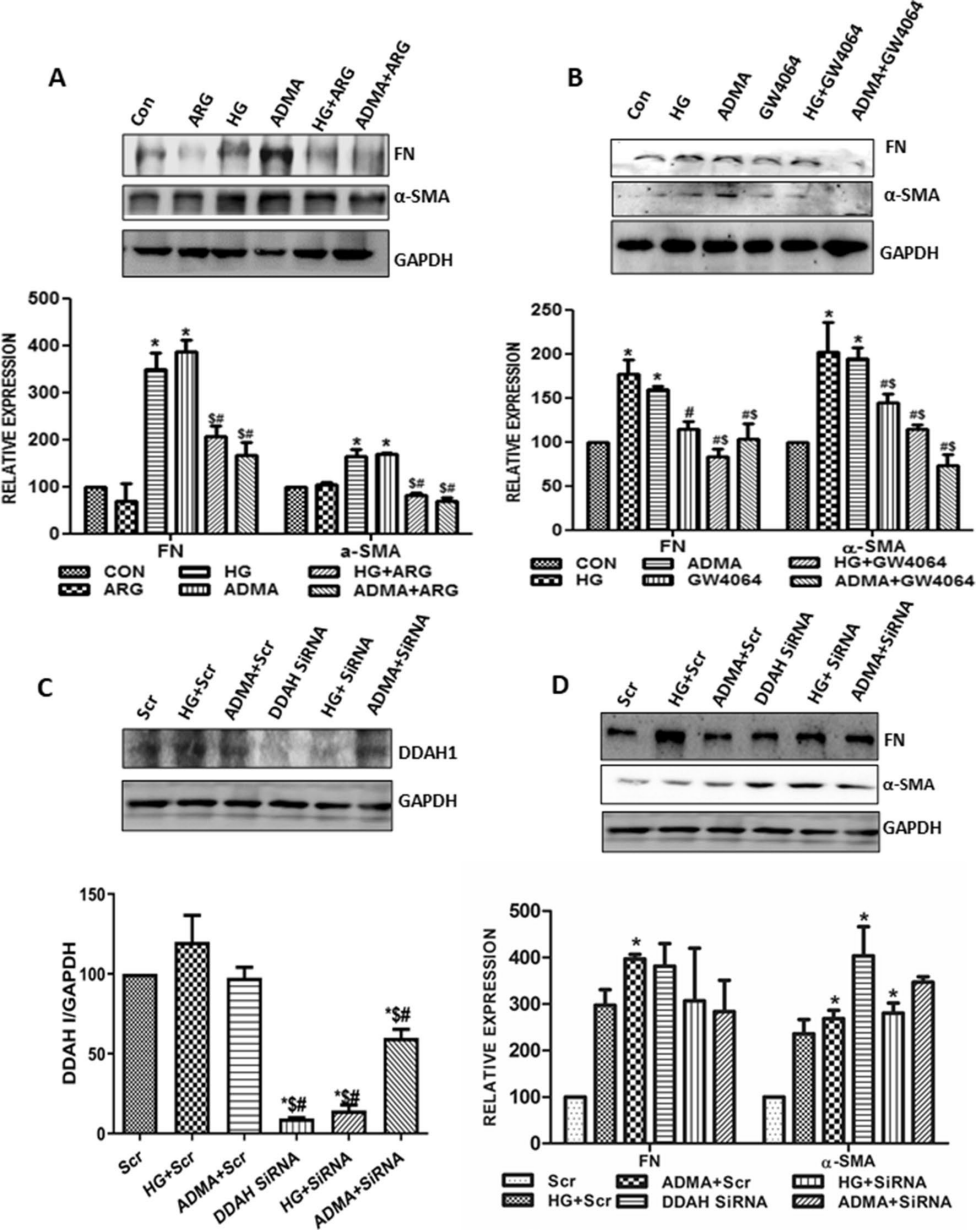
ADMA regulates the expression of fibrotic markers under high glucose condition: (**A**,**B**) Cells were pretreated with ARG/GW4064 for 30 min before HG/ADMA stimulation and were harvested after 24 h treatment. Fibronectin and α-SMA were detected by immunoblotting with GAPDH as loading control. (**C,D**) Cells were transfected with DDAHl siRNA(siRNA)/Scramble RNA(Scr). Two days after the transfection, cells were treated with HG/ADMA and were harvested after 24 h treatment. DDAH1, fibronectin and α-SMA were detected by immunoblotting with GAPDH as loading control. Band intensity was calculated using the ImageJ software. Results are presented as Mean ± SD of three independent experiments. *p < 0.05 compared to the Control, ^#^p < 0.05 compared to the HG, ^$^p < 0.05 compared to the ADMA. Original uncropped images of the blots are given in Supplementary Fig. S6.

To further study the independent role of ADMA in the kidney cell injury, we inhibited ADMA by pre-treating the cells with l-Arginine or GW4064 or selectively knocking down the DDAH 1. Pre-treatment of GW4064 significantly increased the DDAH1 expression (Supplementary Fig. S4). Pre-treatment with l-Arginine (Fig. 3A) and GW4064 (Fig. 3B) in HG/ADMA stimulated NRK49-F cells showed significant reduction in the relative expression levels of differentiated fibrosis markers. To confirm the role of ADMA in the myofibroblast activation, we specifically silenced DDAH 1 (Fig. 3C) and then we studied the relative expression levels of myofibroblast activation markers. They were significantly increased in the DDAH 1 silenced renal cells treated with ADMA or HG compared to the cells treated with ADMA and HG alone (Fig. 3D). From these results, we could conclude that ADMA regulates fibrosis in an independent manner.

Taken together, ADMA and HG increased cell migration, proliferation and extra cellular matrix proteins and augment myofibroblast activation, leading to the primary cause for the fibrosis whereas l-arginine and GW4064 restored the effect of ADMA induced cell injury. This signifies the independent role of ADMA in causing kidney fibrosis under high glucose milieu.

### ADMA increases intracellular ROS generation via up-regulation of NOX4

**Figure 4:**
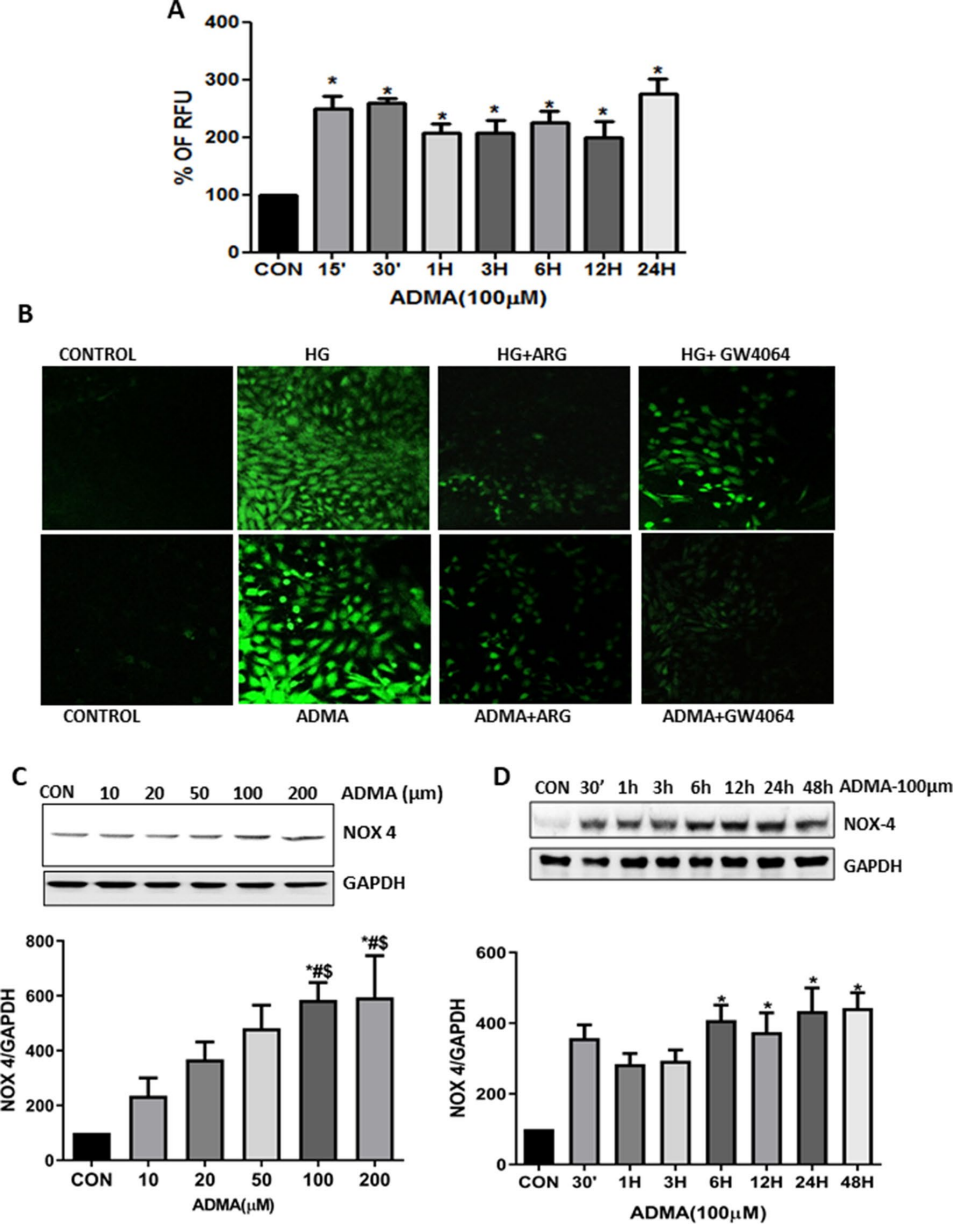
ADMA increases intracellular ROS generation via up-regulation of NOX4: (**A**) Cells were treated with ADMA in time dependent manner and intracellular ROS generation was analysed by multimode reader. The data are represented as % of Relative Fluorescence Unit (RFU). (**B**) Representative photomicrographs of intracellular ROS generation in cells pretreated with ARG/GW4064 for 30 min before HG/ADMA stimulation. Cells were treated with ADMA in (**C**) dose and (**D**) time dependent manner and were harvested after respective conditions. NOX4 was detected using immunoblot with GAPDH as loading control. Results are presented as Mean ± SD of three independent experiments. Band intensity was calculated using the ImageJ software.*p < 0.05 compared to the Control. ^#^p < 0.05 compared with ADMA (10 μM), ^$^p < 0.05 compared with ADMA (20 μM). Original uncropped images of the blots are given in Supplementary Fig. S7.

To check the engagement of ADMA with HG in the activation of fibrosis pathway, we studied the effect of ADMA on the ROS generation. As shown in the Fig. 4A, ADMA significantly increased the ROS generation in a time dependent manner. Furthermore, we examined whether l-Arginine or GW4064 can neutralize the ADMA induced ROS generation. The renal cells pre-treated with l-Arginine or GW4064 significantly blocked the ROS generation (Fig. 4B).

NADPH oxidase is a major source of ROS generation in renal cells. As we observed that ADMA increases the ROS generation, we then analysed NOX4 expression, the predominant isoform of NADPH oxidase in the kidney, under ADMA treatment in the renal fibroblasts. We found that ADMA increased NOX4 expression significantly in a dose and time-dependent manner (Fig. 4C,D). From this we confirmed that in the cultured renal fibroblasts cells, ADMA elicits a rapid increase in the NOX4 expression levels, which is associated with increased intracellular ROS production.

DPI has been considered to be an effective inhibitor against all NOX isoforms. We inhibited NOX4 by pre-treating the cells with DPI (10 µM) and then treated with HG/ADMA in the renal cells. We found that NOX4 inhibition significantly decreased the HG/ADMA induced ROS generation. This experiment shows the involvement of NOX4 in the ADMA/HG induced ROS generation (Supplementary Fig. S5).

### ADMA augments myofibroblast activation through NOX-4

**Figure 5:**
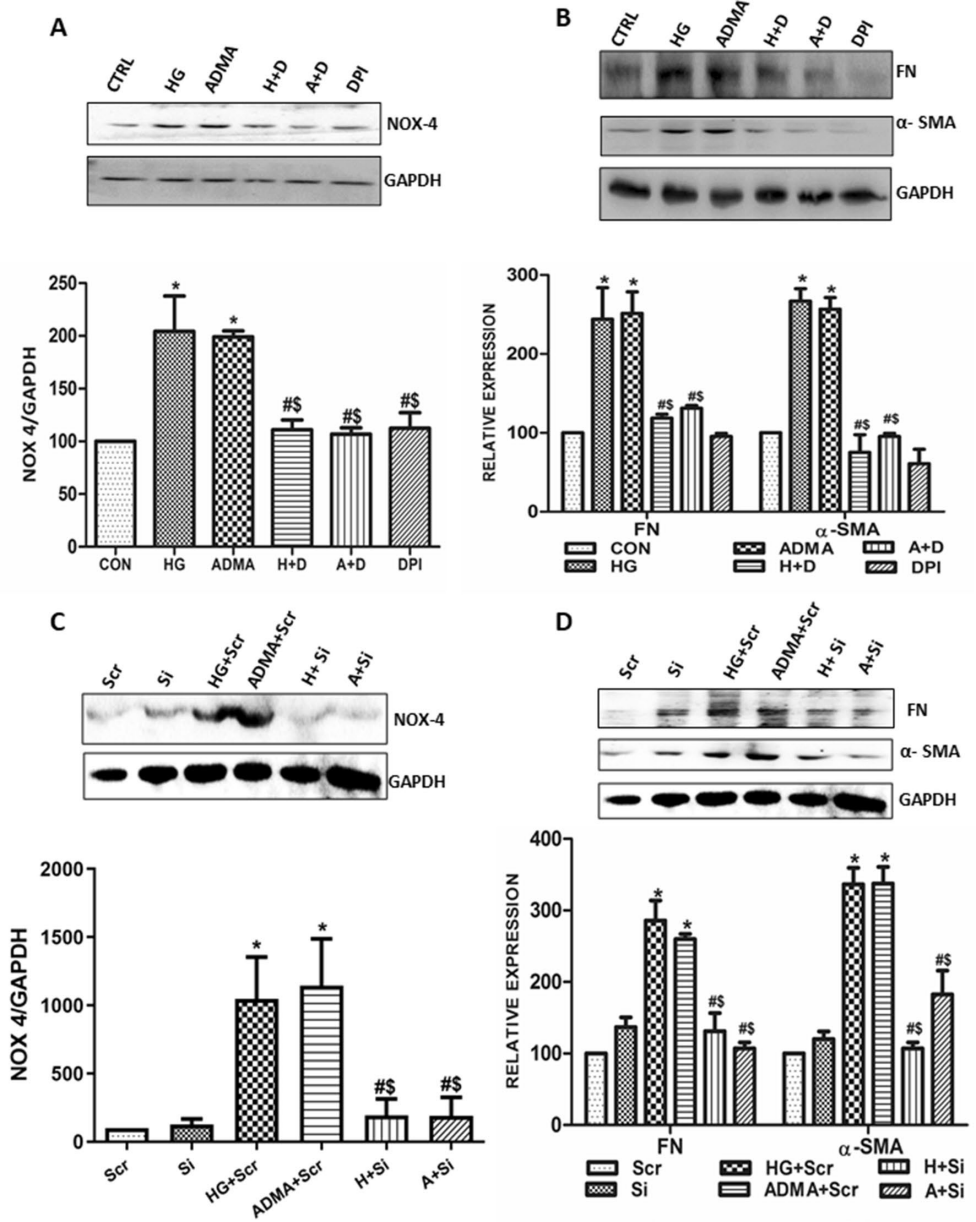
ADMA augments myofibroblast activation through NOX4: (**A**,**B**) Cells were pretreated with DPI(D) for 30 min before HG(H)/ADMA(A) treatment and were harvested after 24 h. NOX4, fibronectin, α-SMA were detected by immunoblot with GAPDH as a loading control. (**C**,**D**) Cells were transfected with NOX4 SiRNA(Si)/Scramble RNA(Scr). Two days after the transfection, cells were treated with HG(H)/ADMA(A) and were harvested after 24 h treatment. NOX4, fibronectin, α-SMA were detected by immunoblot with GAPDH as a loading control. Band intensity was calculated using the ImageJ software. Results are presented as Mean ± SD of three independent experiments. *p < 0.05 compared to the Control, ^#^p < 0.05 compared to the HG, ^$^p < 0.05 compared to the ADMA. Original uncropped images of the blots are given in Supplementary Fig S8.

We speculated that ADMA induced NOX-4 derived ROS generation could be involved in the early events of diabetic kidney disease such as the extracellular matrix accumulation. To confirm this, we inhibited NOX4 by pre-treating the cells with DPI. As expected, expression levels of NOX4 were significantly reduced in the cells pre-treated with DPI (Fig. 5A). Further, We found that HG/ADMA stimulated expression levels of extra cellular matrix proteins fibronectin and α-SMA were also significantly reduced by the pre-treatment with DPI (Fig. 5B).

As our results needs to be strengthened by further analysis, we inhibited the NOX-4 by transfecting the cells with NOX4 siRNA (Fig. 5C), as DPI also acts as an inhibitor of other ROS sources. As anticipated, NOX-4 siRNA transfected cells treated with HG/ADMA showed significantly reduced expression of fibronectin and α-SMA (Fig. 5D).

These findings demonstrated that ADMA induced NAD(P)H oxidase-4 is involved in the HG stimulated ECM proteins fibronectin and α-SMA production in the renal fibroblasts cells.

### ADMA induced myofibroblast activation is characterized by stimulation of ERK1/2 pathway

**Figure 6:**
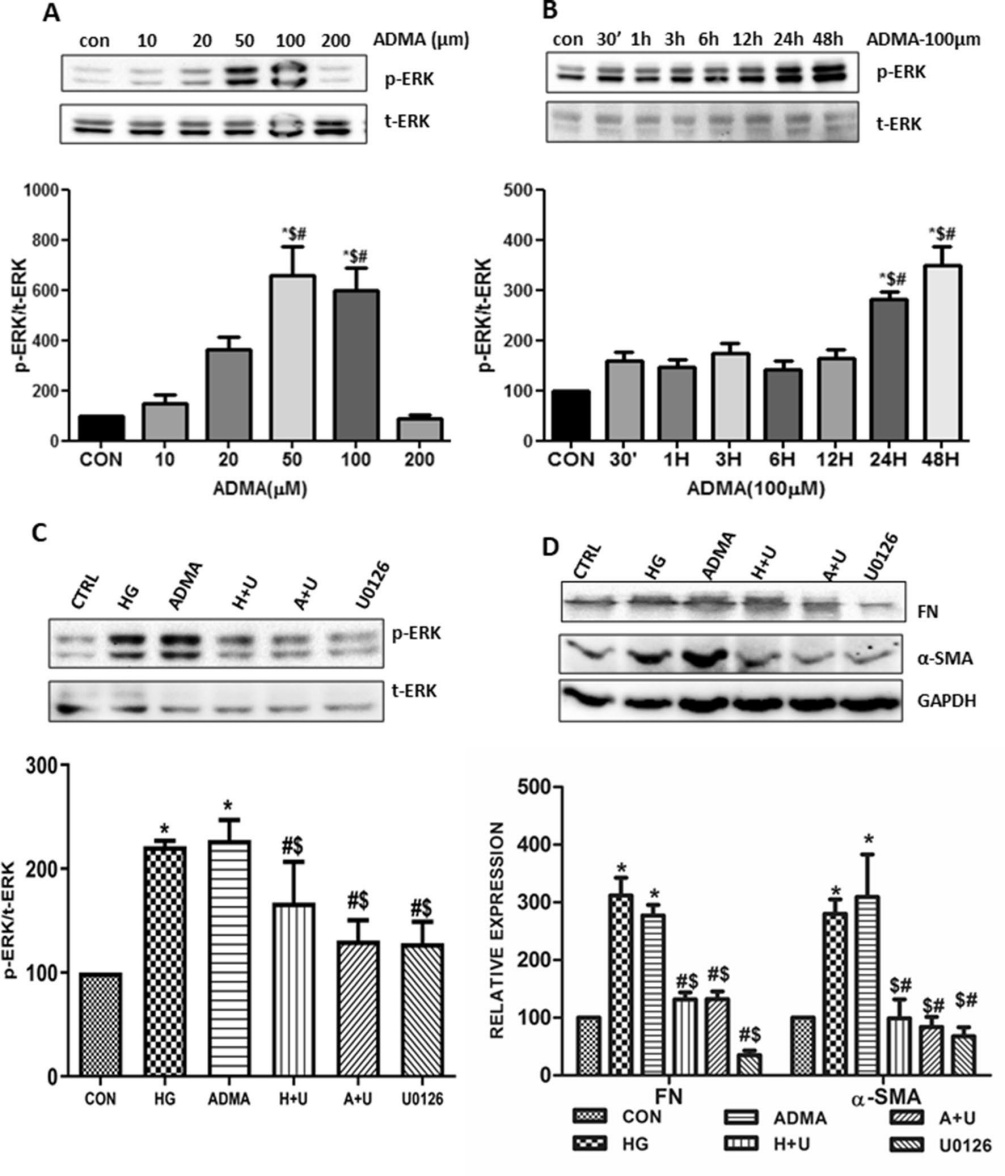
ADMA induced myofibroblast activation is characterized by stimulation of ERK1/2 pathway: cells were treated with ADMA in (**A**) dose and (**B**) time dependent manner and were harvested after respective conditions. p-ERK was detected by using immunoblot with t-ERK as loading control. (**C**,**D**) Cells were pretreated with U0126(U) for 30 min before HG(H)/ADMA(A) treatment and were harvested after 24 h. p-ERK, fibronectin and α-SMA were detected by immunoblot. Band intensity was calculated using the ImageJ software. Results are presented as Mean ± SD of three independent experiments. (**A**) *p < 0.05 compared with Control, ^#^p < 0.05 compared with ADMA (10 μM), ^$^p < 0.05 compared with ADMA (20 μM). (**B**) *p < 0.05 compared with Control, ^$^p < 0.05 compared with 30′, ^#^p < 0.05 compared with 1 h. (**C**,**D**) *p < 0.05 compared to the Control, ^#^p < 0.05 compared to the HG, ^$^p < 0.05 compared to the ADMA. Original uncropped images of the blots are given in Supplementary Fig. S9.

MAP kinases such as ERK1/2 have a central role in the fibrosis pathway which is stimulated by ROS. Since ADMA stimulated ROS generation, we were interested to study the role of ADMA in ERK1/2 signaling pathway. We found that HG/ADMA significantly and progressively increased the phosphorylation of ERK1/2 in a dose and time kinetic manner (Fig. 6A,B).

To further elucidate the role of ADMA in ERK1/2 signaling pathway in the process of fibrosis, we studied the expression levels of extra-cellular matrix proteins in the HG/ADMA treated renal cells pre-treated with UO126, ERK inhibitor. Expression levels of fibronectin and α-SMA were significantly decreased in the UO126 pre-treated cells (Fig. 6C,D). These results suggest that ADMA is involved in the activation of MAP kinase ERK1/2 leading to the kidney cell fibrosis.

### ADMA regulates NOX4/ROS/ERK signaling pathway during the high glucose induced myofibroblast activation

**Figure 7:**
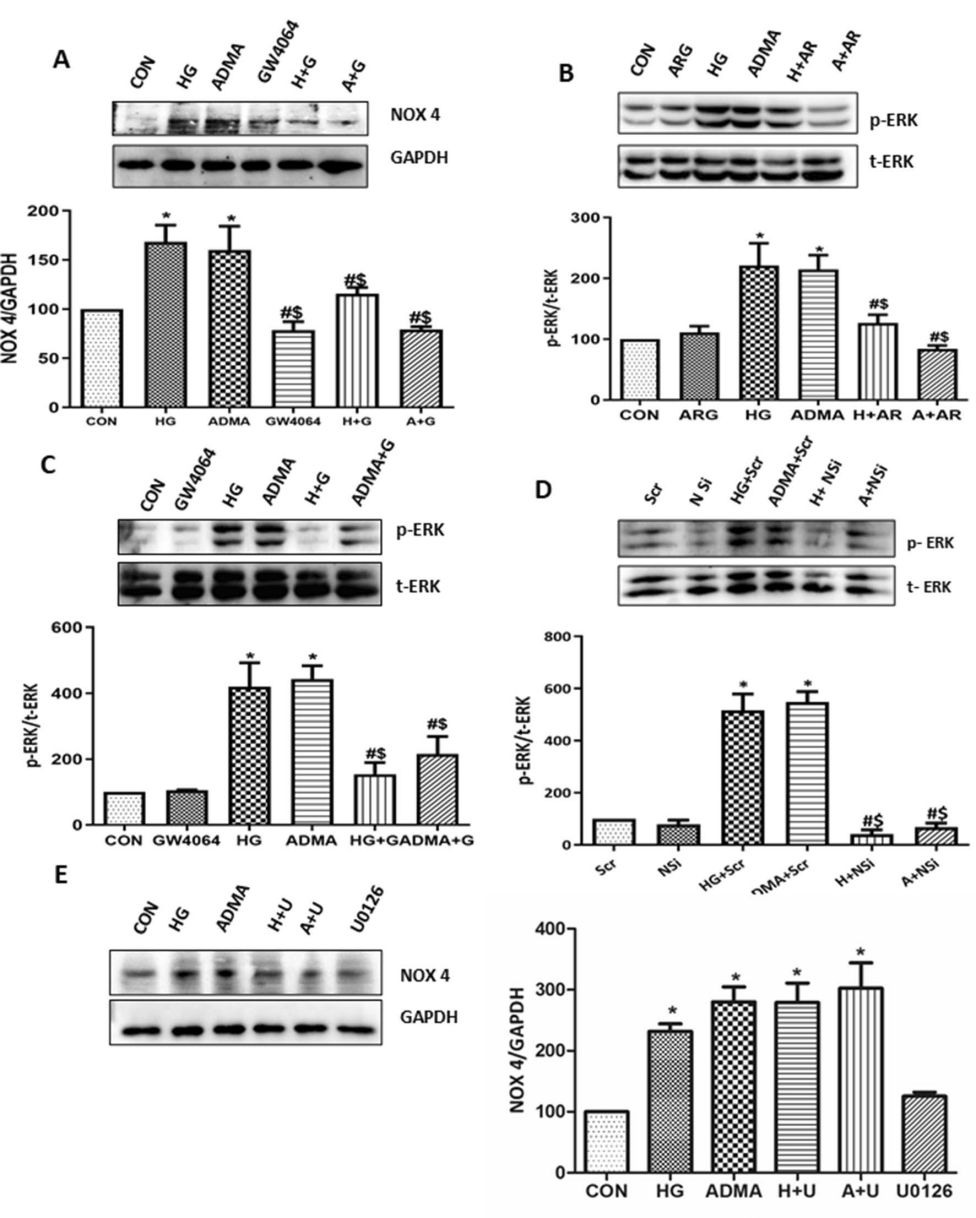
ADMA regulates NOX4/ROS/ERK signaling pathway during the high glucose induced myofibroblast activation: (**A**,**C**) Cells were pretreated with GW4064(G) for 30 min before HG(H)/ADMA(A) treatment and were harvested after 24 h. (**B**) Cells were pretreated with l-Arginine **(**ARG/AR) for 30 min before HG/ADMA treatment and were harvested after 24 h. (**D**) Cells were transfected with NOX4 SiRNA (N Si)/Scramble RNA (Scr). Two days after the transfection, cells were treated with HG(H)/ADMA(A) and were harvested after 24 h treatment. (**E**) Cells were pretreated with U0126(U) for 30 min before HG(H)/ADMA(A) treatment and were harvested after 24 h. p-ERK and NOX4 of respective conditions were detected by using immunoblot with t-ERK and GAPDH as loading control respectively. Band intensity was calculated using the ImageJ software. Results are presented as Mean ± SD of three independent experiments. *p < 0.05 compared to the Control, ^#^p < 0.05 compared to the HG, ^$^p < 0.05 compared to the ADMA. Original uncropped images of the blots are given in Supplementary Fig. S10.

To dissect out the complex signaling mechanisms of ADMA induced fibrosis, we analysed the expression levels of NOX4 in the HG/ADMA stimulated cells pre-treated with GW4064. The GW4064 pre-treatment significantly reduced the expression levels of NOX4 in the HG/ADMA stimulated cells (Fig. 7A).

To determine whether the activation of ERK 1/2 pathway is ADMA dependent and consequently via NOX4, we analysed the phosphorylation of ERK1/2 in the cells treated with ARG/GW4064/NOX4SiRNA (Fig. 7B–D). The respective treatments significantly reduced the HG/ADMA induced phosphorylation of ERK1/2. This implies that NOX4 is an activator of ERK1/2. We also found that inhibiting ERK1/2 did not significantly affect the expression levels of NOX4 (Fig. 7E). These results clearly emphasize the role of NOX4 as an upstream activator of ERK1/2 in the ADMA induced kidney cell fibrosis. They also demonstrated that the central role of ADMA in the renal fibrosis is mediated through NADPH oxidase-ERK1/2 signaling pathways.

### ADMA induced myofibroblast activation and its effect on ROS-NOX4-ERK signaling axis in mesangial cells

**Figure 8:**
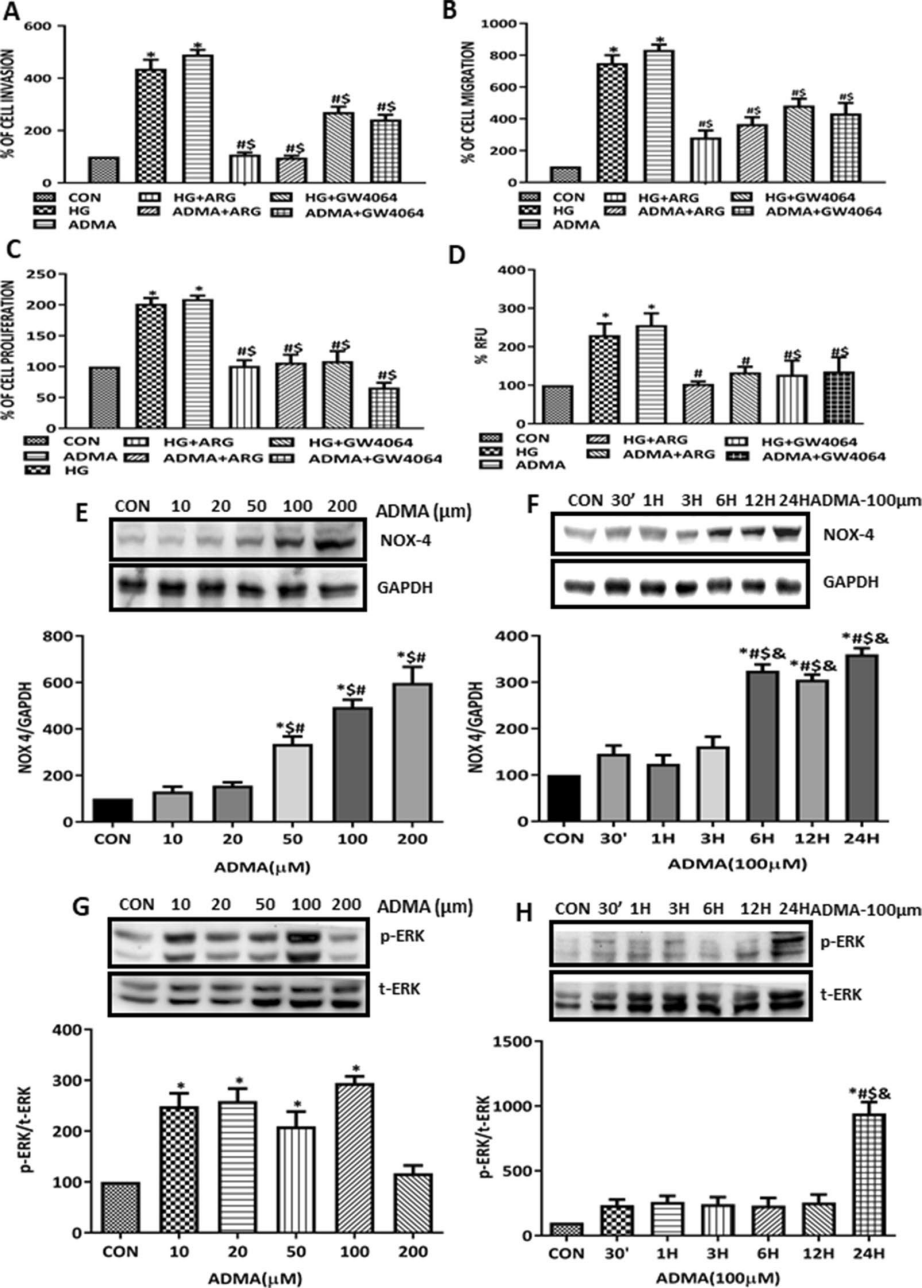
ADMA induced myofibroblast activation and its effect on ROS-NOX4-ERK signaling axis in rat mesangial cells: (**A**) RMC cells pre-treated with ARG/GW4064 for 30 min and then treated with HG/ADMA for 10 h in transwell and the pictures were taken in four random high power fields. Cells were counted from four random microscopic fields per insert in triplicate. The migrated cell numbers were normalized to that of the control group. The results were expressed as % of invasion. (**B**) RMC monolayers pre-treated with ARG/GW4064 for 30 min were scratched by using pipette and treated with HG/ADMA for 12 h, the percentage of wound width was calculated and the results were expressed as % of migration compared to control. (**C**) Cell proliferation was determined by BrdU incorporation assay, and the results were expressed as % of proliferation compared with control. (**D**) Cells pretreated with ARG/GW4064 for 30 min before HG/ADMA treatment and intracellular ROS generation was analysed by multimode reader. The data are represented as % of Relative Fluorescence Unit (RFU). Cells were treated with ADMA in (**F**,**H**) time and (**E**,**G**) dose dependent manner and cells were harvested after respective conditions. NOX-4, p-ERK and t-ERK of respective conditions were detected by immunoblot with GAPDH as loading control. Results are presented as Mean ± SD of three independent experiments. (**A**–**D**) *p < 0.05 compared to the Control, ^#^p < 0.05 compared with HG, ^$^p < 0.05 compared with ADMA. (**E**,**G**) *p < 0.05 compared with Control, ^$^p < 0.05 compared with ADMA (10 μM), ^#^p < 0.05 compared with ADMA (20 μM). (**F**,**H**) *p < 0.05 compared with Control, ^#^p < 0.05 compared with 30′, ^$^p < 0.05 compared with 1H, ^&^p < 0.05 compared with 3H. Original uncropped images of the blots are given in Supplementary Fig. S11.

Beyond fibroblasts, mesangial cells have been additionally considered as an precursor for myofibroblast cell type. Hence, in this study we analysed the effect of ADMA on the Rat Mesangial cells. We found that ADMA as well as HG significantly increased the events of myofibroblast activation. Inhibition of ADMA attenuated the HG and ADMA induced myofibroblast activation such as cell invasion (Fig. 8A), migration (Fig. 8B) and proliferation (Fig. 8C). We also studied the effect of ADMA on the intracellular ROS generation, we found that ADMA significantly increased the ROS generation and upon inhibition with l-arginine and GW4064 intracellular ROS generation was significantly reduced (Fig. 8D). Further to delineate the role of ADMA in the NOX4/ERK signaling axis in the mesangial cells we performed the time and dose kinetics. We found that ADMA significantly increased NOX4 and ERK activity in dose (Fig. 8E,G) and time dependent manner (Fig. 8F,H). From these observation we confirmed that ADMA plays as an myofibroblast activator causing the kidney fibrosis by ensuing ROS-NOX4-ERK signaling axis.

## Discussion

By impairing the production of nitric oxide, ADMA contributes to the development of vascular malfunctioning^16,17^. Many studies have reported that elevated ADMA concentration in the circulation is a potential biomarker for the prediction of cardiovascular diseases. We found that high glucose significantly upregulated the mRNA expression of PRMT1 and decreased AGXT2 expression in renal cells which may lead to the accumulation of ADMA.

From our previous study, we demonstrated that circulatory levels of ADMA were increased in the diabetic kidney disease and can be used as a potential biomarker for the prediction of diabetic kidney disease at an early stage^8^. But there are limited studies on causal role of ADMA in the pathogenesis of renal diseases^18–20^. In our present study we are delineating the molecular mechanisms by which ADMA causes kidney cell injury under high glucose condition. The nut-shell findings from our study are as follows:

(i) ADMA causes/stimulates fibrotic events such as cell migration, invasion, proliferation and expression of differentiated fibrotic markers.
(ii) ADMA induces ROS formatioan under high glucose condition. The primary source of ADMA induced ROS is via NAD(P)H oxidase-4 in the kidney cells.
(iii) ADMA induced ROS generation further activates the ERK-1/2 pathway leading to fibrosis.

**Figure 9:**
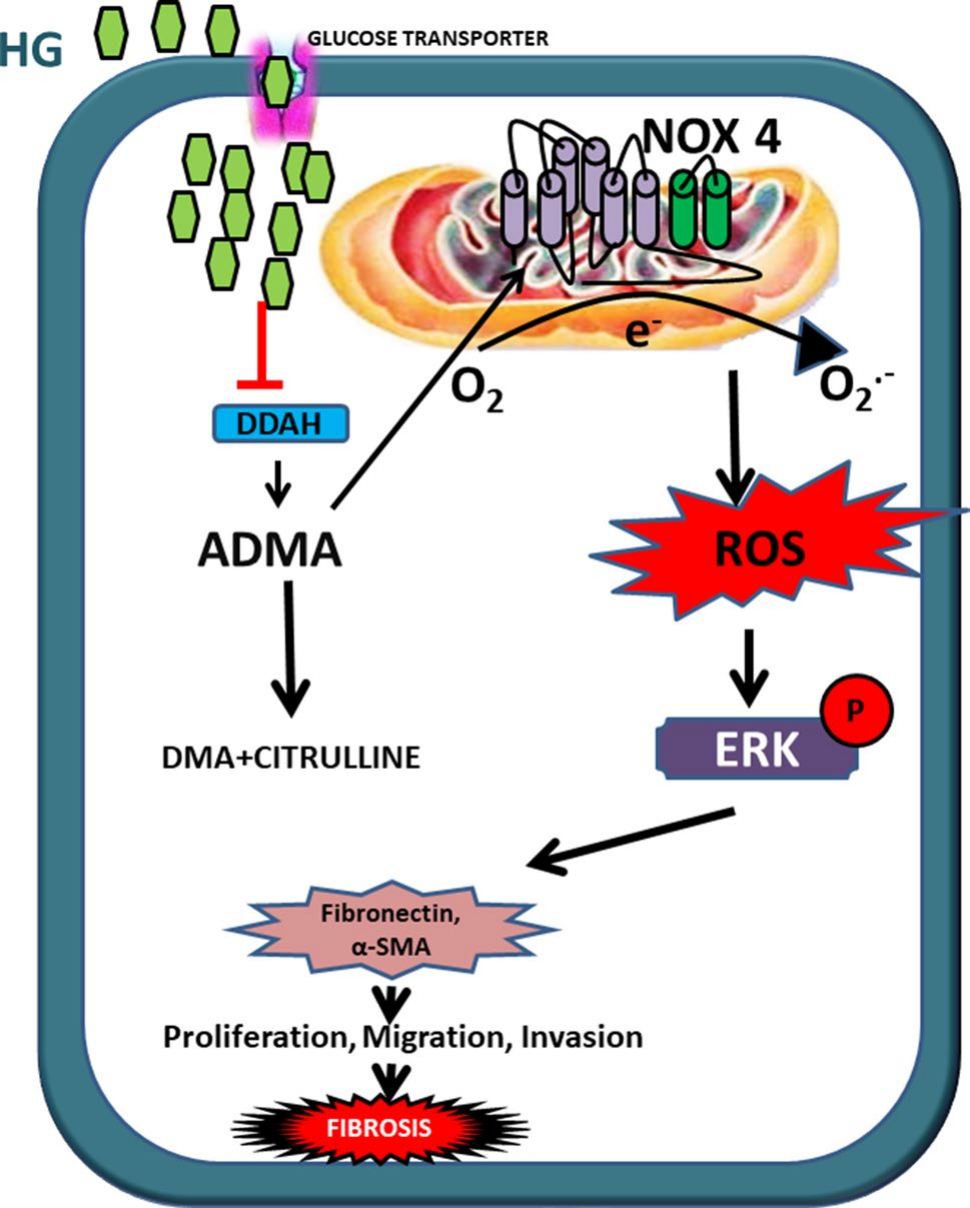
The schematic representation of molecular mechanism of ADMA induced renal cell fibrosis under high glucose condition.

While our study is the first report to demonstrate ADMA as a causative factor of kidney cell injury in high glucose condition, we have also delineated the molecular signaling pathway by which ADMA induces renal cell fibrosis. The schematic representation (Fig. 9) of our findings explains the fibrotic effect of ADMA via up-regulation of ROS/NOX-4/ERK1/2 signaling in cells under high glucose exposure. This complex pathway leads to the myofibroblast activation causing the renal cell fibrosis.

Fibrosis is an irreversible damage to the renal parenchyma due to several patho-physiological conditions mainly diabetes^21,22^, hypertension, cardiovascular disease^23,24^, etc., where the damaged tissues are replaced with the scarring connective tissues. Fibrosis is an inevitable event leading to the diabetic kidney disease. There are several pathways involved in the pathogenesis of fibrosis. Each pathway is regulated by a stimulus and other downstream regulators. Previous studies have suggested that myofibroblast activation acts as a major contributor to the renal fibrosis as they are the major producer of ECM proteins^25,26^.

These myofibroblasts originates from the resident fibroblasts, mesangial cells, fibrocytes, and tubular epithelial cells. The cells especially fibroblasts and mesangial cells, are activated and proliferated during the persistent high glucose condition^27,28^. But how high glucose alone stimulates renal fibrosis is still a mystery. There are other products which are also involved in the high glucose induced renal cell injury.

ADMA, an endogenous inhibitor of nitric oxide, is synthesized during post translational modification and involved in the many patho-physiological conditions. In this present study, we report that ADMA acts as one of the primary stimuli for the activation of fibrotic pathway under hyperglycaemic condition. Inhibition of DDAH activity by high glucose treatment will lead to the alteration of ADMA metabolism and causes cell injury. DDAH overexpression in the rat remnant kidney model has been shown to ameliorate proteinuria, tubulointerstial fibrosis and also inhibit upregulation of TGF-β expression^29^. While DDAH-2 overexpression in mice has been shown to inhibit iopromide-induced kidney injury, DDAH-2 knockdown was also shown to aggravate the iopromide induced acute kidney injury^30^. Ischaemia/reperfusion diminished DDAH activity leading to the accumulation of ADMA in the kidney^31^. Several reports also suggested the role of DDAH1 & 2 in the regulation of glucose and insulin metabolism^32–34^. But in contradictory, Rodionov et al. observed that ADMA elevation does not exacerbate development of diabetic retinopathy in mice with streptozotocin-induced diabetes^35^. This could be due to the in-built limitation in the STZ-induced model of diabetic nephropathy as well as other mechanisms that translates the genesis and progression of renal disease. However, there are now growing body of evidences suggesting the role of DDAH and ADMA in the pathophysiology of diabetic nephropathy. In a recent study, Ad-h-DDAH-1 treatment in diabetic mice reversed not only albuminuria and histological changes but also reduced glomerular macrophage recruitment, inflammatory cytokine and fibrotic markers, kidney ADMA levels, and oxidative stress via ADMA-NOS3 interaction^36^. One of our earlier studies also provided an association of elevated levels of circulatory levels of ADMA in patients with diabetic nephropathy and found decreased DDAH activity under high glucose treatment^8^. Thus, we confirm that DDAH plays a major role in the metabolism of ADMA.

Further, we reported that the circulatory levels of nitric oxide were proportionately reduced with the increased levels of ADMA in diabetes patients with albuminuria and thus we reported a negative correlation between ADMA and nitric oxide levels^8^. These studies and the results of the present manuscript emphasize a role of ADMA in the NOS and nitric oxide regulation. As we saw significantly decreased expression levels of phospho eNOS in cells treated with ADMA, it implies that ADMA induced pro-fibrotic process is mediated through alterations in NOS activity. This study is the first to elucidate the mediators involved in the ADMA induced fibrotic pathway.

Oxidative stress, imbalance in the generation and degradation of reactive oxygen species, considered to be a major player in the process of fibrosis. Increased evidence suggests that oxidative stress and ROS are involved in the generation and activation of fibrosis by increasing cell migration, transition, and proliferation by activating various signaling cascades^37^. NAD(P)H oxidase family, which includes seven isoforms, have low constitutive activity under physiological conditions. Upon deleterious stimuli these isoforms are upregulated. It has been well documented that NOX4 expression in the kidney is related with the development and progression of kidney fibrosis, particularly in diabetic condition^38^. Here we observed that ADMA significantly increased intracellular ROS generation in the renal fibroblasts in time-dependent manner through NOX4 stimulation. It has been well established that NOX4 is a major source of ROS under disease condition. NOX-4, predominantly expressed in the kidney is playing a major role in the kidney fibrosis^39^. Consistent with this, we showed that ADMA up regulated the NOX-4 expression in the renal fibroblasts cells. Renal fibroblasts play a dominant role in the renal fibrosis and they responded to ADMA with the increase in the intracellular ROS generation through NOX-4. We also shown that ADMA induced fibrotic process is inhibited in the NOX-4 siRNA transfected renal cells and DPI treated cells.

ROS generated via NOX-4 activates various patho physiological signaling pathways. It has been reported that ROS has a strong ability to activate ERK signaling cascade leading to renal fibrosis under hyperglycaemic condition^40–42^. ERK signaling controls the primary events such as cellular proliferation, migration and invasion. Under pathophysiological conditions it has been documented that persistent activation of ERK leads to myofibroblast activation^43,44^. We confirmed the activation of ERK1/2 by ADMA in time and dose dependent manner. Further, we have also delineated that ADMA induced ERK1/2 activation leads to myofibroblast activation. From these findings, we demonstrate holistically that ADMA activates NOX-4, which is an upstream activator of ERK1/2 signaling cascade, leading to renal fibrosis.

Further, there are several glucose transporters including GLUT transporters which regulate glucose homeostasis and there is an increasing attention to the investigations on the sodium-glucose cotransporter (SGLT) inhibitors which represent a new alternative for treating patients with diabetes mellitus and its vascular complications^45^. Canagliflozin (an inhibitor of SGLT2) has been demonstrated to mitigate kidney dysfunction and to attenuate eGFR decline and albuminuria, emphasizing a nephroprotective effect of this drug^46^. Therefore, we suggest that future studies should investigate the effect of SGLT inhibitors to see whether the beneficial renal protection could occur via inhibition of intracellular ADMA accumulation and/or ADMA pathway.

Collectively, our study implies that ADMA involves in the initiation and progression of renal cell fibrosis by activating NOX-4/ROS/ERK1/2 signaling pathway (Fig. 9). These results reveal new mechanistic link between ADMA and kidney cell injury. It is important for further study to look for strategic methods to prevent or reverse pathophysiological manifestation of kidney fibrosis under high glucose condition. Our findings suggest that ADMA could be a potential therapeutic target in preventing diabetic kidney injury.

## Supplementary information

Supplementary Information
