# Supplementary material for: Asymmetric dimethylarginine (ADMA) accelerates renal cell fibrosis under high glucose condition through NOX4/ROS/ERK signaling pathway
Source: Sci Rep. 2020 Sep 29;10:16005. doi: 10.1038/s41598-020-72943-2 (PMC7525240; doi:10.1038/s41598-020-72943-2)

Asymmetric Dimethylarginine (ADMA) accelerates renal cell fibrosis under high glucose condition through NOX4/ROS/ERK signaling pathway

Isaivani Jayachandran, Saravanakumar Sundararajan, Saravanakumar Venkatesan, Sairaj Paadukaana, Muthuswamy Balasubramanyam, Viswanathan Mohan, Nagaraj Manickam*

Department of Vascular Biology, Madras Diabetes Research Foundation & Dr. Mohan's Diabetes Specialities Centre, WHO Collaborating Centre for Non-communicable Diseases Prevention and Control &ICMR Center for Advanced Research on Diabetes, Chennai, India

Correspondence:

*** Nagaraj Manickam;** nagaraj@mdrf.in

Supplementary Fig S1: Effect of high glucose on PRMT1 in NRK-49F cells

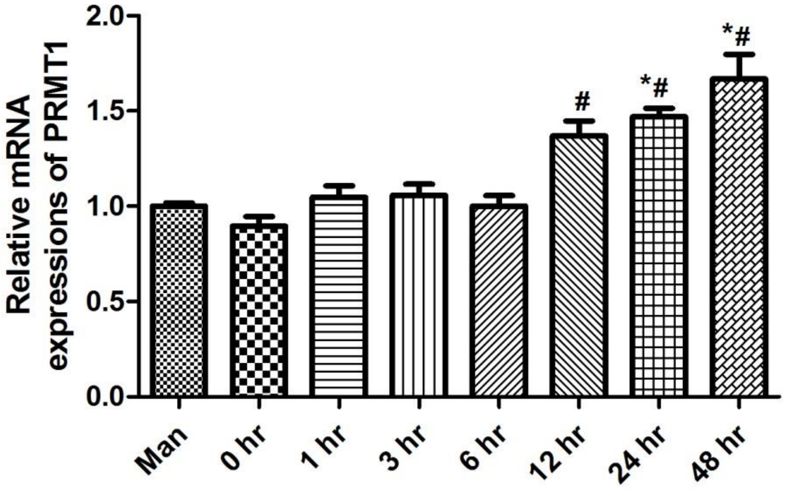

Supplementary Figure.S1: Cells (NRK-49F) were treated with high glucose (25.5 mM) at indicated time points. Relative mRNA expression of PRMT1 was detected. All values are represented as Mean ± SEM. * p<0.05 compared to Mannitol; # p< 0.05 compared to 0-hr (HG),

Supplementary Fig. S2: Effect of high glucose on AGXT2

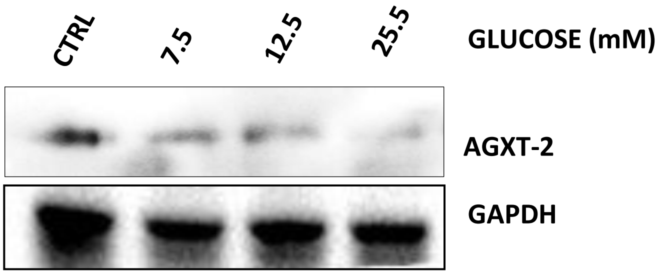

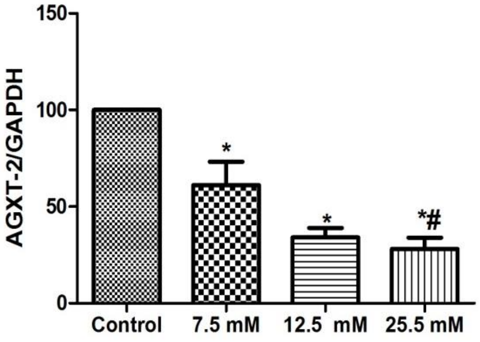

Supplementary Figure S2: Fibroblast cells (NRK-49F) were treated with high glucose in dose dependent manner for 24 hrs. AGXT2 and GAPDH were detected by immunoblot. Band intensity was calculated using the ImageJ software * $p < 0.05$ compared to Control, # $p < 0.05$ compared to 7.5 mM Glucose. (Original Uncropped blot is given as Supplementary Fig. S12)

Supplementary Fig.S3: ADMA induced renal cell fibrosis is dependent on eNOS activity:

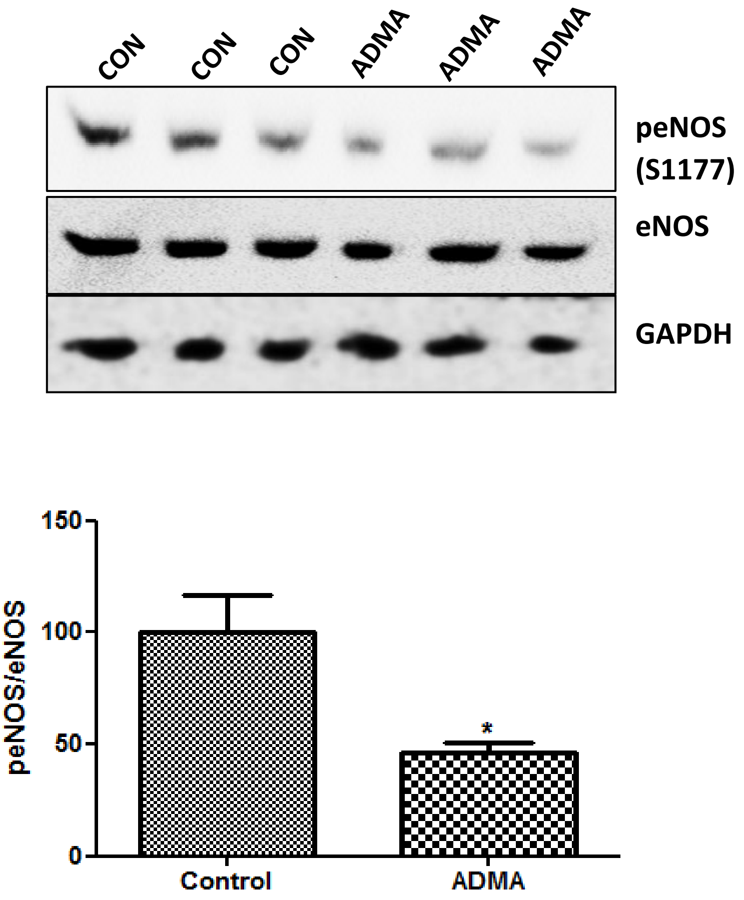

Supplementary Figure.S3: Cells (NRK-49F) were treated with ADMA (100µM) for 24 hours and harvested. Cell lysates were used to immunoblot, peNOS, eNOS and GAPDH. Band intensity was calculated using the ImageJ software * $p < 0.05$ compared to Control. (Original Uncropped blot is given as Supplementary Fig. S13)

Supplementary Fig.S4: Effect of GW4064 on DDAH1 expression

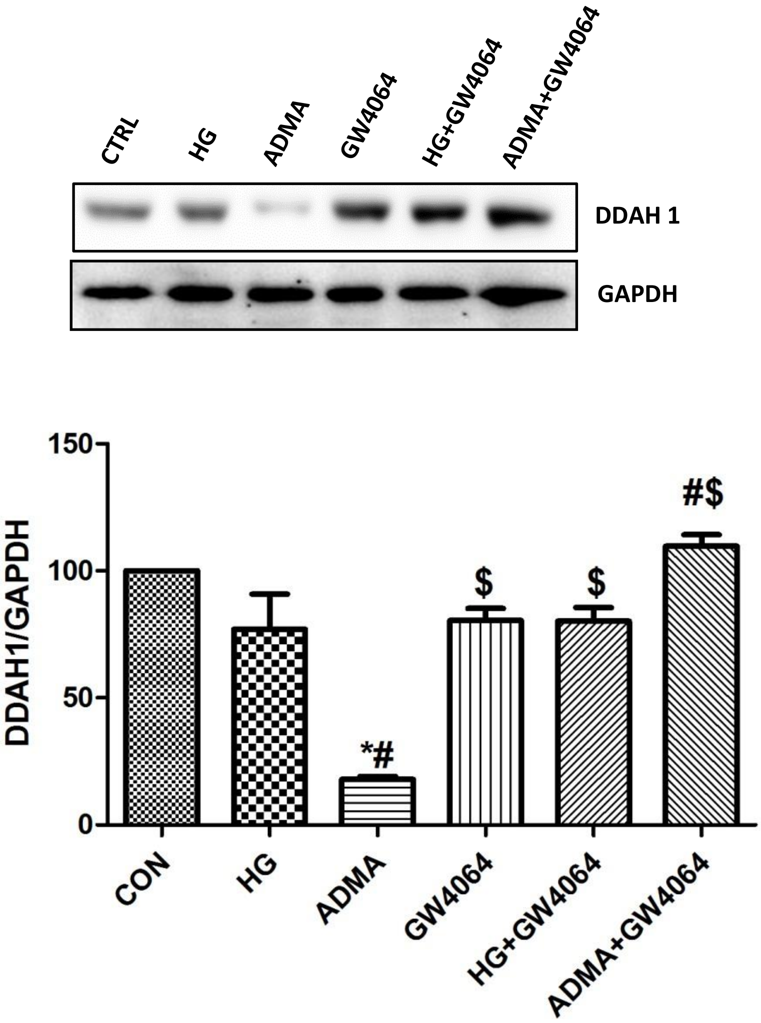

Supplementary Figure.S4: NRK-49 F cells were pre-treated with GW4064 and incubated with HG (25.5 mM of Glucose) and ADMA (100 µM) for 24 hours, and then the cells lysates were probed for DDAH1, GAPDH antibodies. Band intensity was calculated using Image J software. All values are represented as Mean± SEM. * $p < 0.05$ compared to Control, # $p < 0.05$ compared to HG, $ $p<0.05$ compared to ADMA. (Original Uncropped blot is given as Supplementary Fig. S14)

Supplementary Fig.S5: ADMA increases intracellular ROS generation via upregulation of NOX4

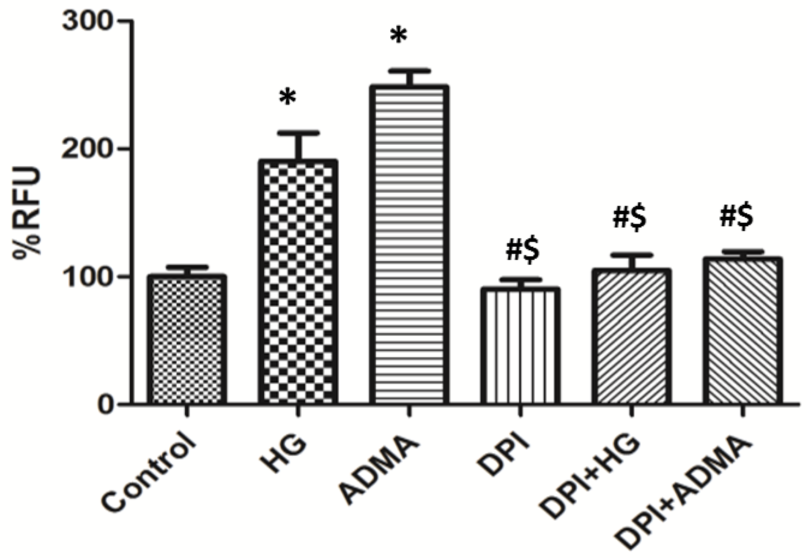

Supplementary Figure.S5: NRK-49F cells were pre-treated with Diphenyleneiodonium (DPI) and incubated with HG (25.5 mM of Glucose) and ADMA (100 µM) for 24 hours. Then the cells lysates were analysed for intracellular ROS accumulation using DCFDA in multimode reader. The data are represented as % of Relative Fluorescence Unit. * $p< 0.05$ compared to Control, # $p< 0.05$ compared to HG, $ $p<0.05$ compared to ADMA.

Supplementary Fig S6: Original Uncropped blot images of Fig 3

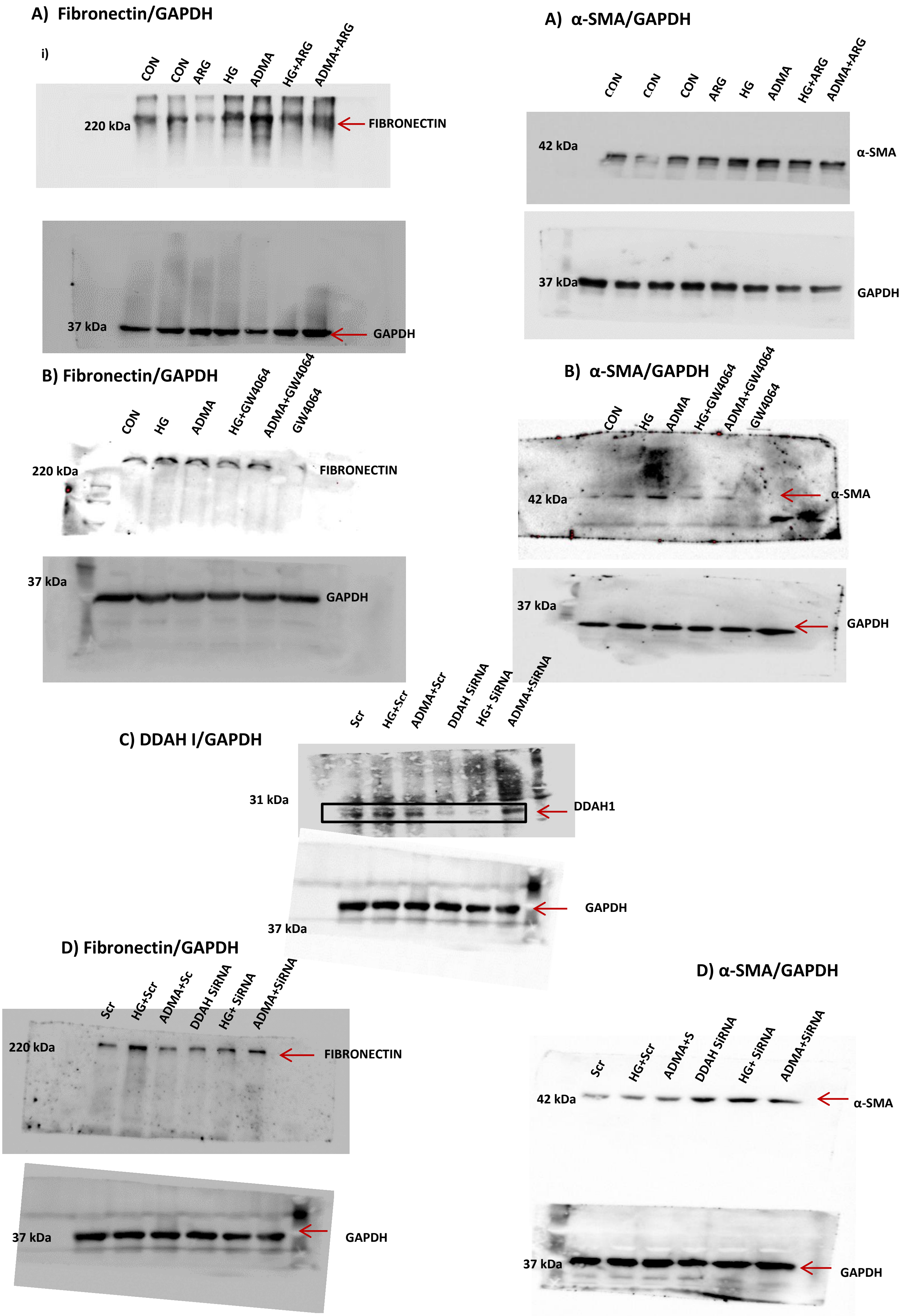

Supplementary Fig.S7: Original Uncropped blot images of Fig 4

C) NOX4/GAPDH

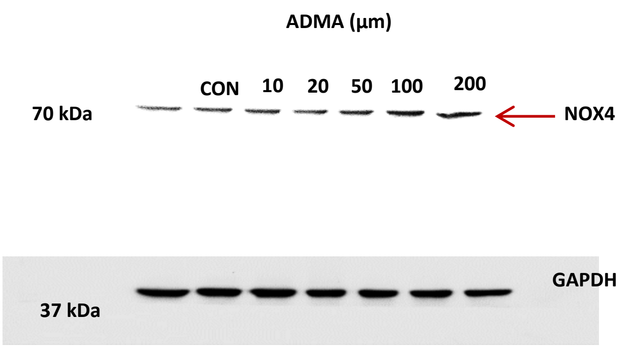

D) NOX4/GAPDH

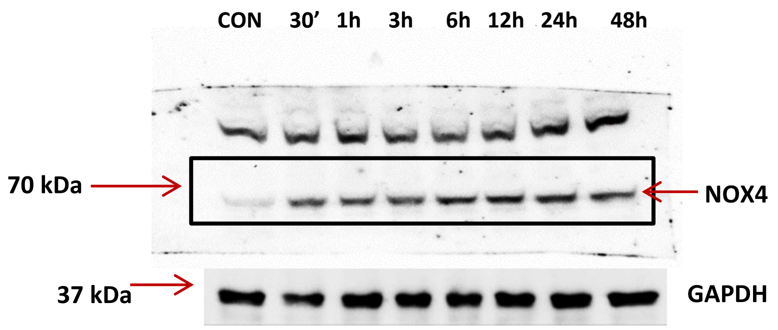

Supplementary Fig S8: Original Uncropped blot images of Fig 5

A) NOX4/GAPDH

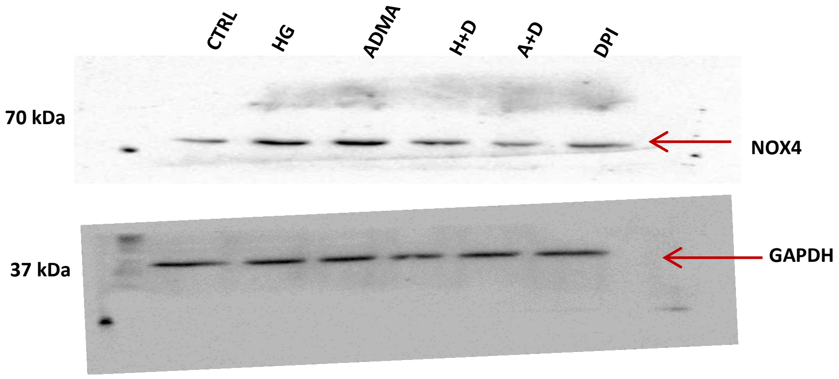

B) Fibronectin/GAPDH

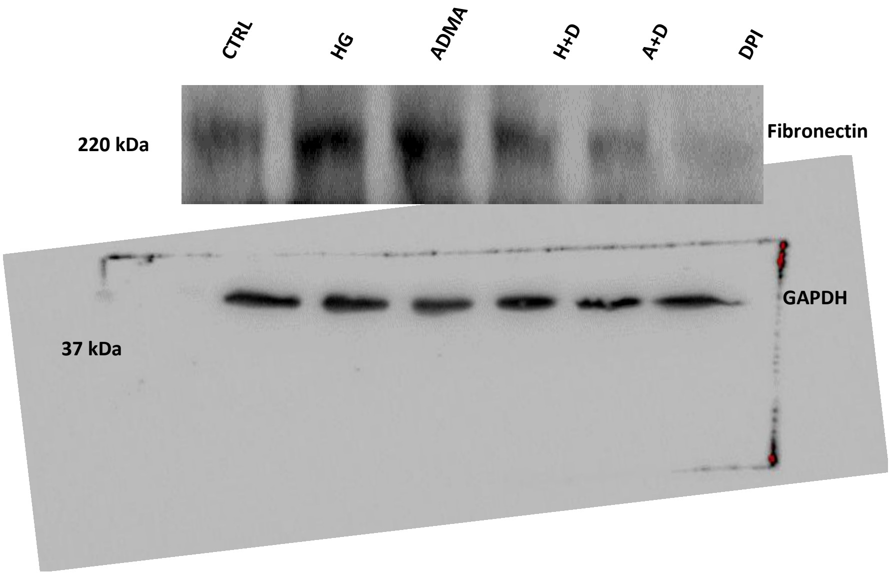

B) α-SMA/GAPDH

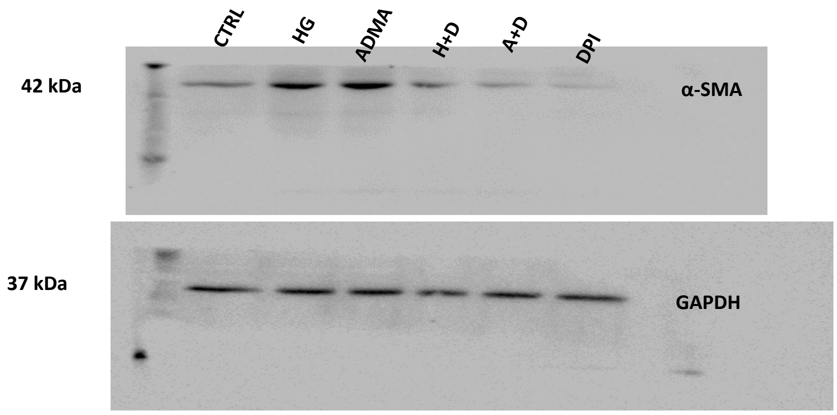

Supplementary Fig.S8: Original Uncropped blot images of Fig 5, continuation

C) Original blots: NOX4/GAPDH

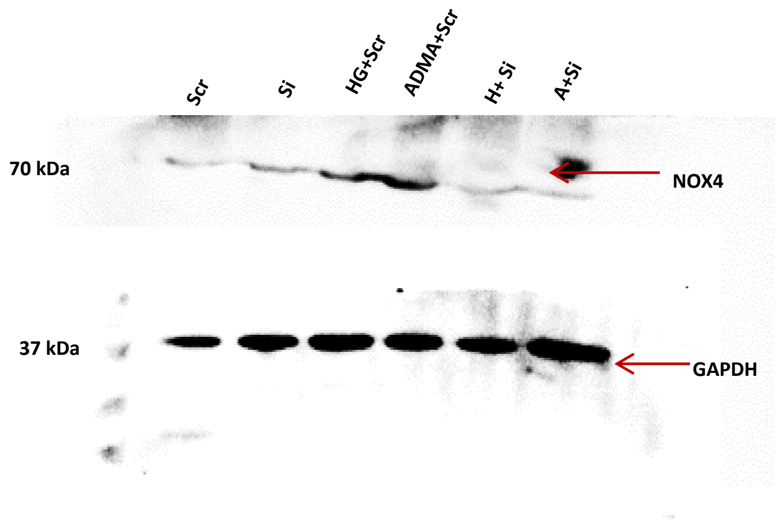

D) Fibronectin/GAPDH

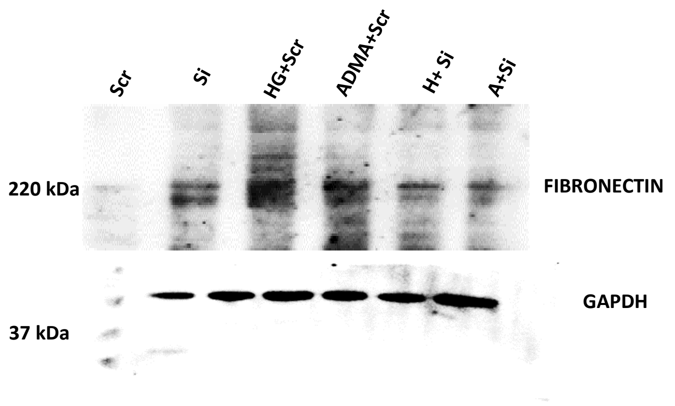

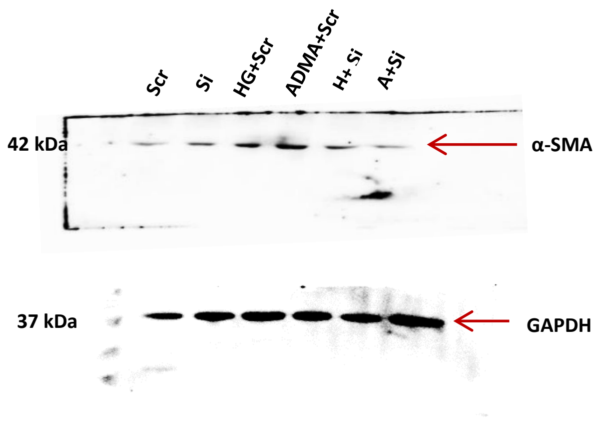

Supplementary Fig.S9: Original Uncropped blot images of Fig 6

A) Original blots: pERK/tERK

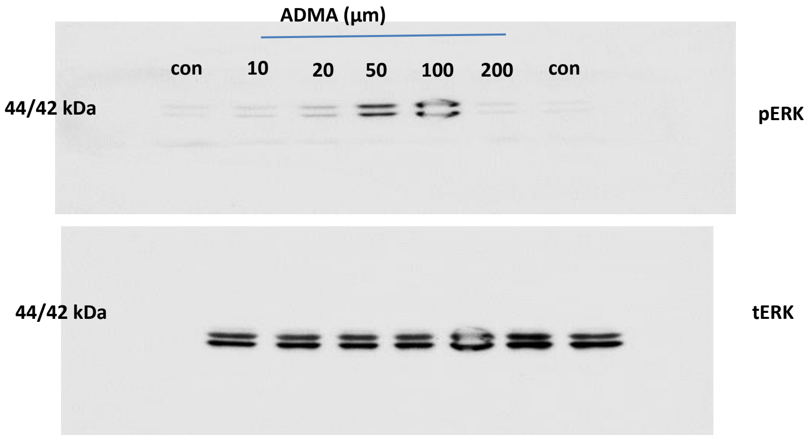

B) Original blots: pERK/tERK

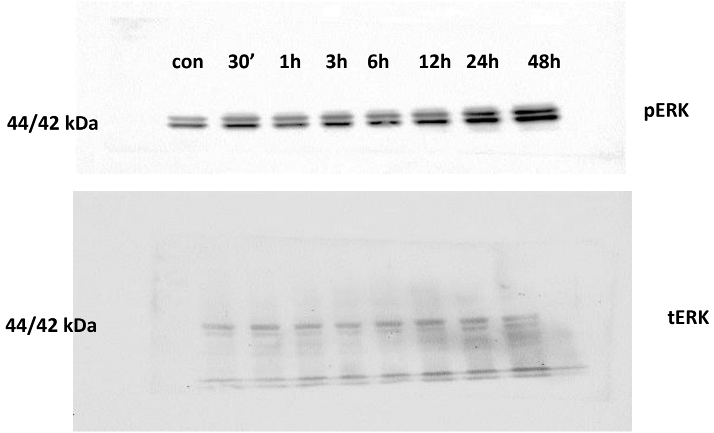

C) Original blots: pERK/tERK

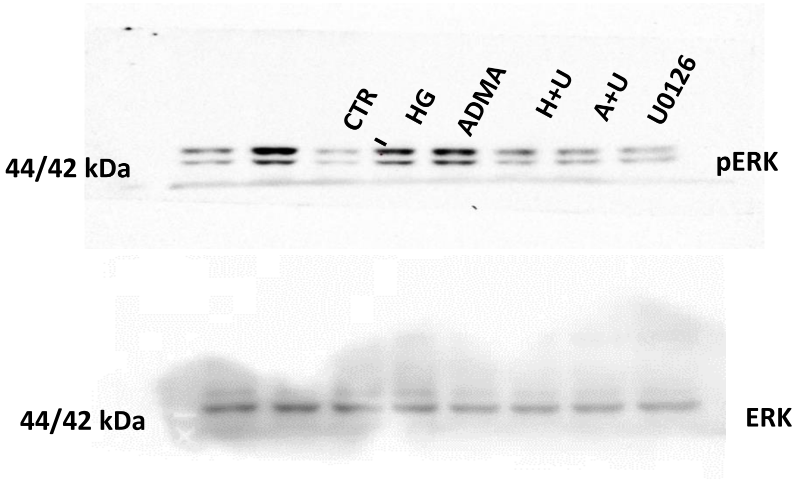

Supplementary Fig.S9: Original Uncropped blot images of Fig 6 Continuation

D) Original blots: Fibronectin/GAPDH

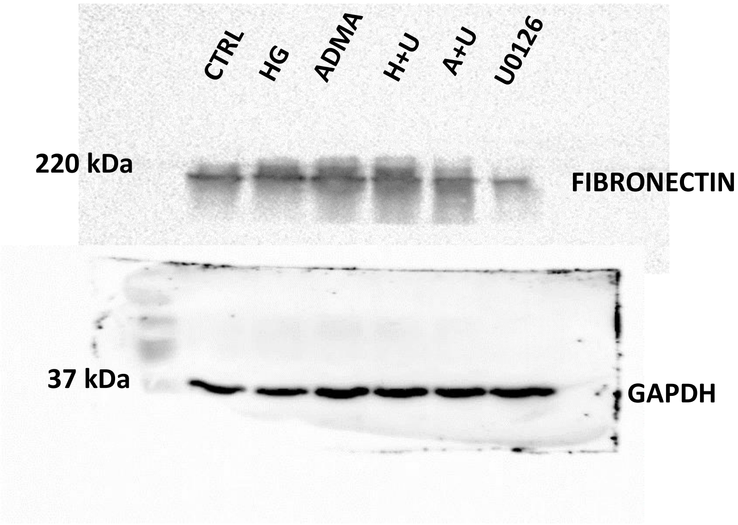

D) Original blots: α-SMA/GAPDH

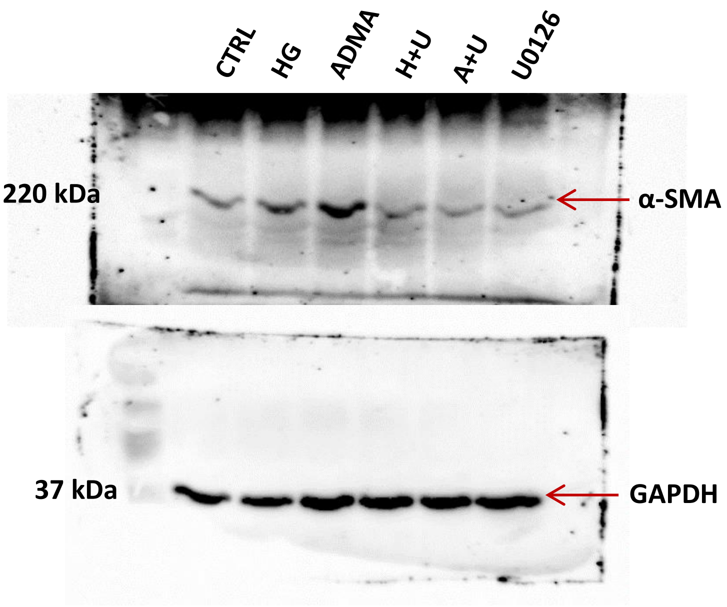

Supplementary Fig.S10: Original Uncropped blot images of Fig 7

A) Original blots: NOX4/GAPDH

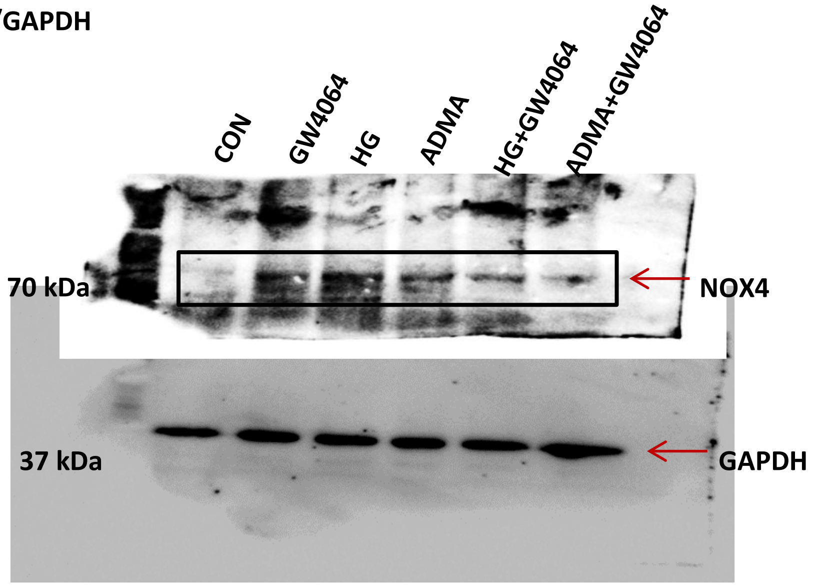

B) Original blots: pERK/tERK

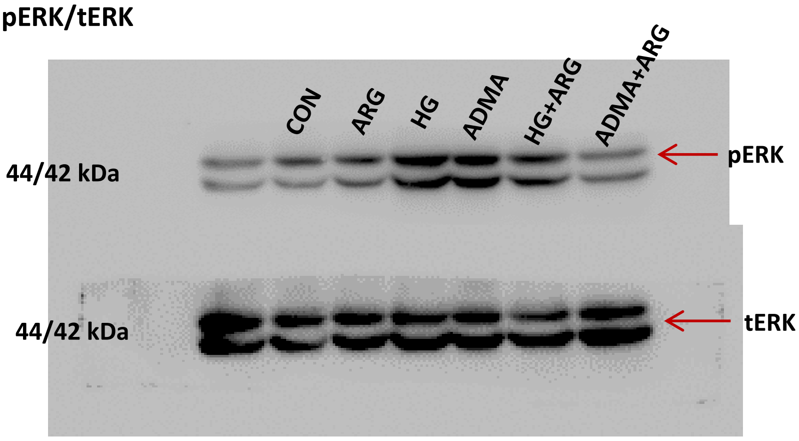

C) Original blots: pERK/tERK

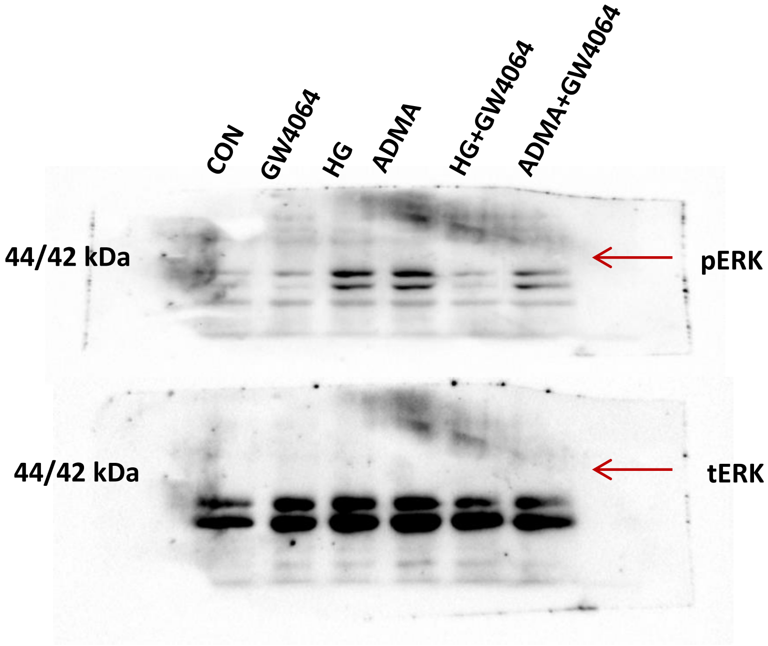

Supplementary Fig.S10: Original Uncropped blot images of Fig 7 Continuation

D) Original blots: pERK/tERK

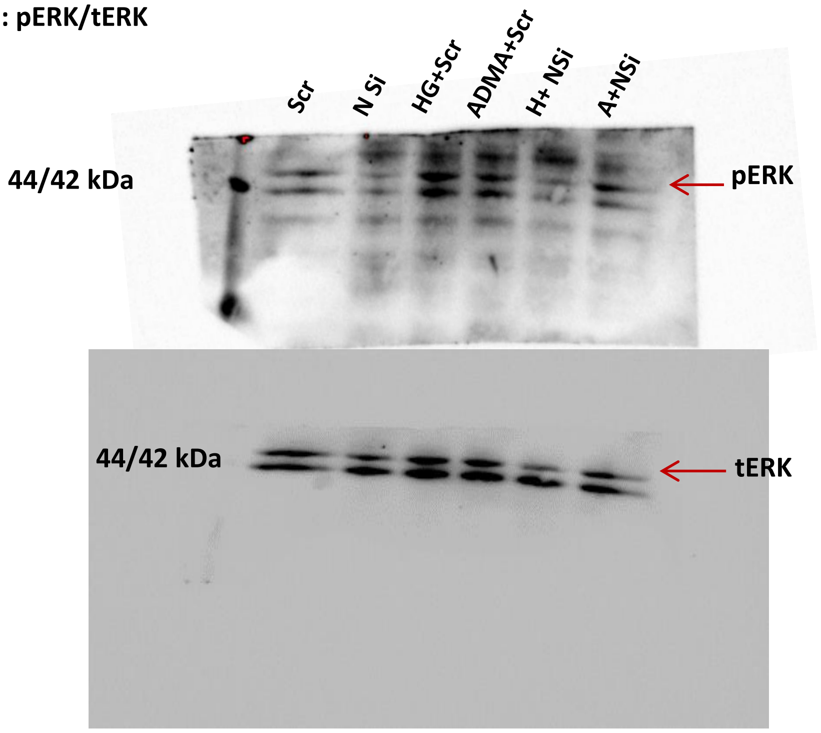

E) Original blots: NOX4/GAPDH

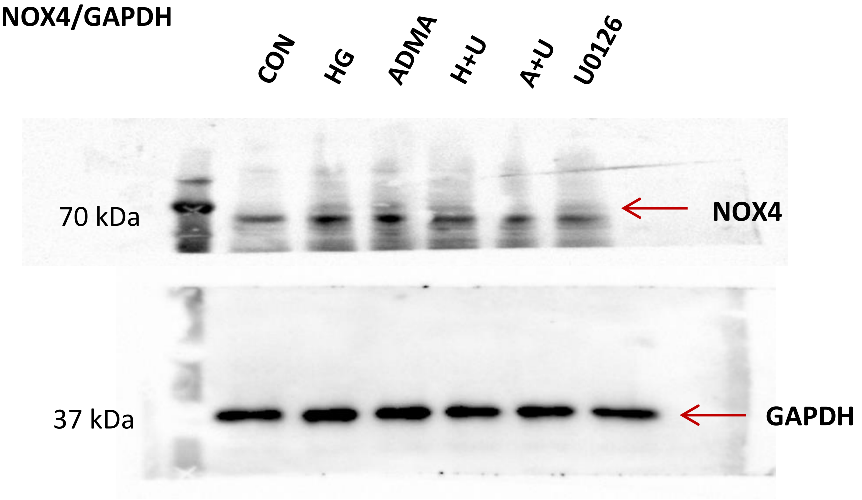

Supplementary Fig.S11: Original Uncropped blot images of Fig 8

E) Original blots: NOX4/GAPDH

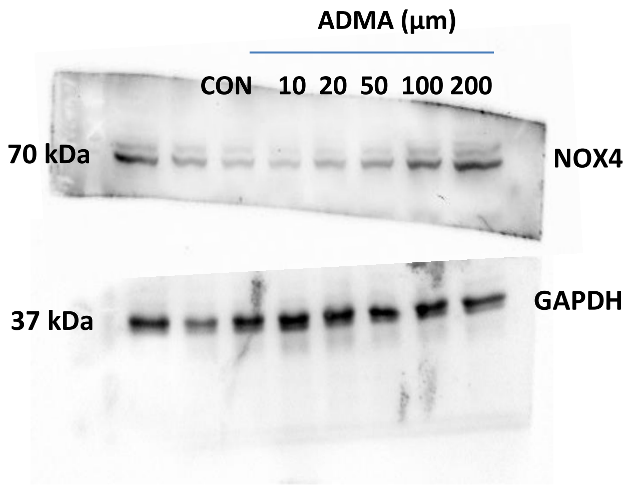

F) Original blots: NOX4/GAPDH

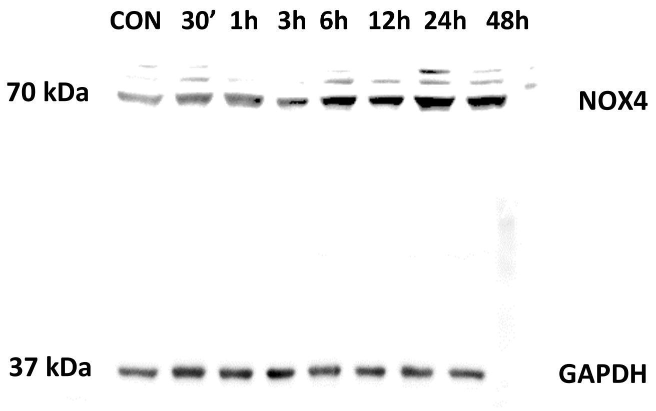

G) Original blots: pERK/tERK

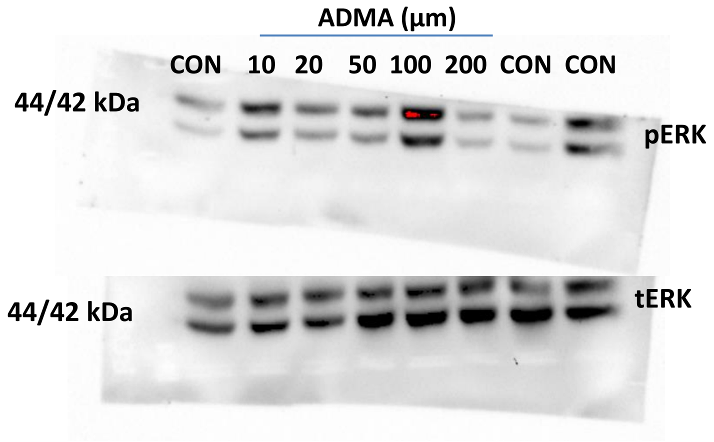

H) Original blots: pERK/tERK

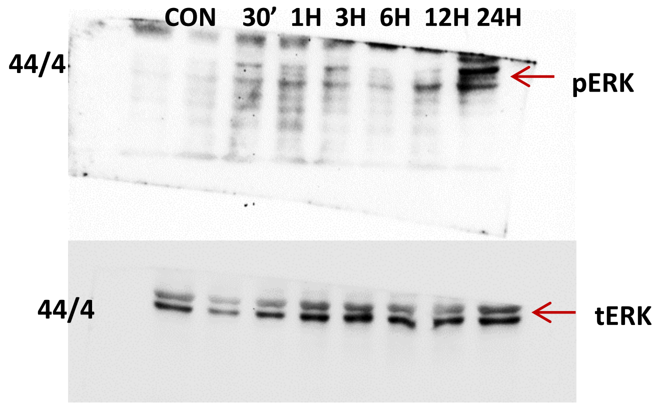

Supplementary Fig.S12: Original Uncropped blot images of Supplementary Fig. S2

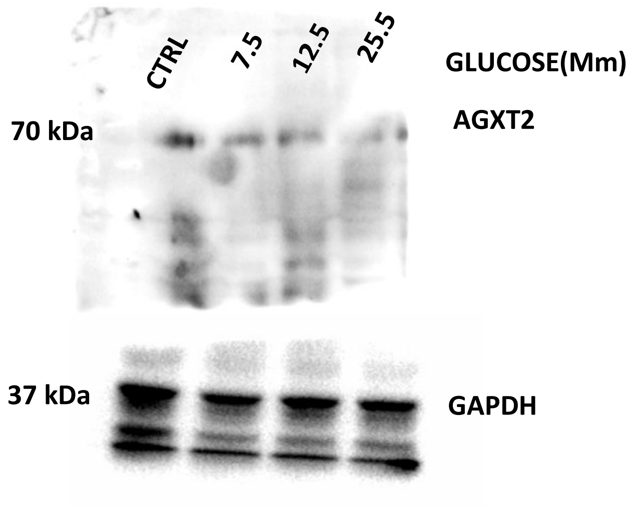

Supplementary Fig.S13: Original Uncropped blot images of Supplementary Fig. S3

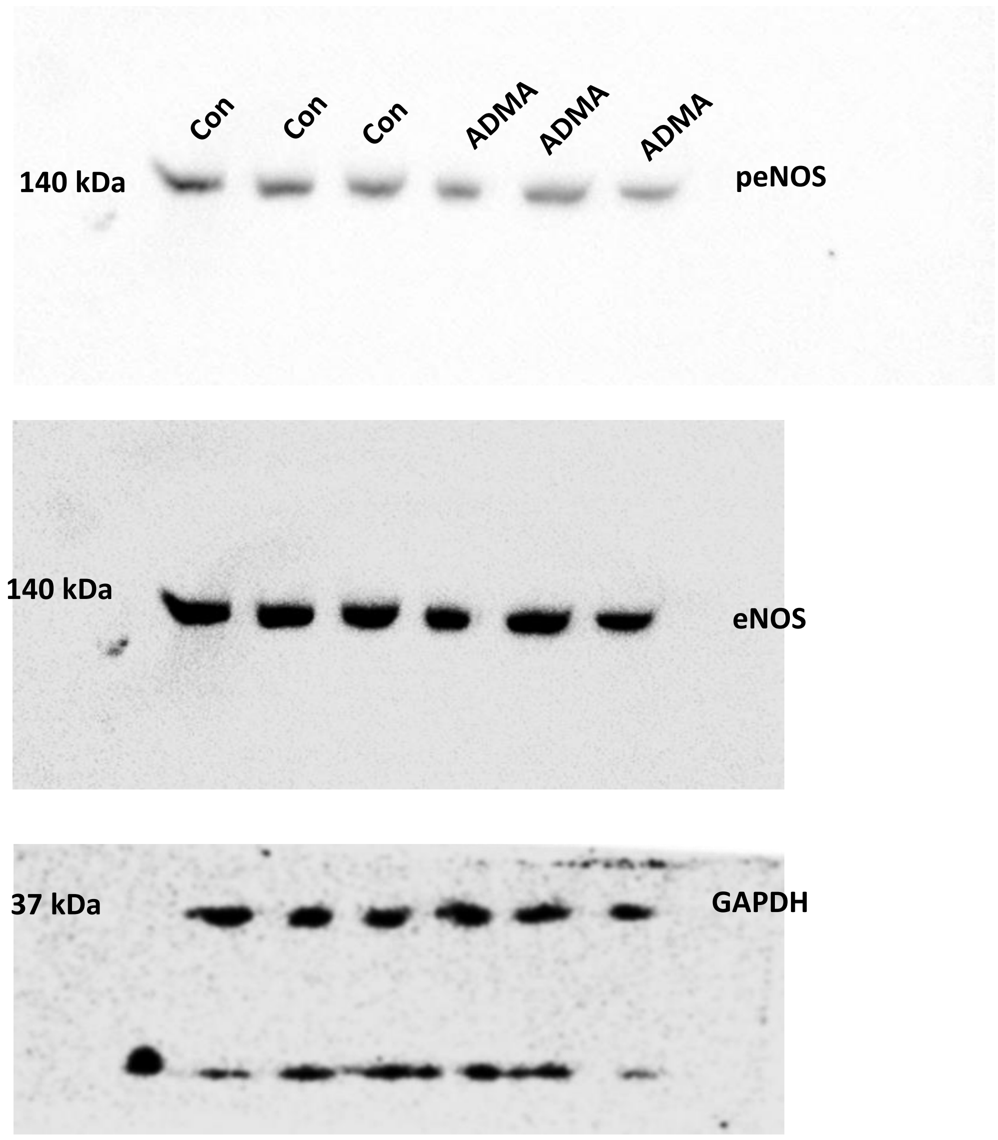

Supplementary Fig.S14: Original Uncropped blot images of Supplementary Fig. S4

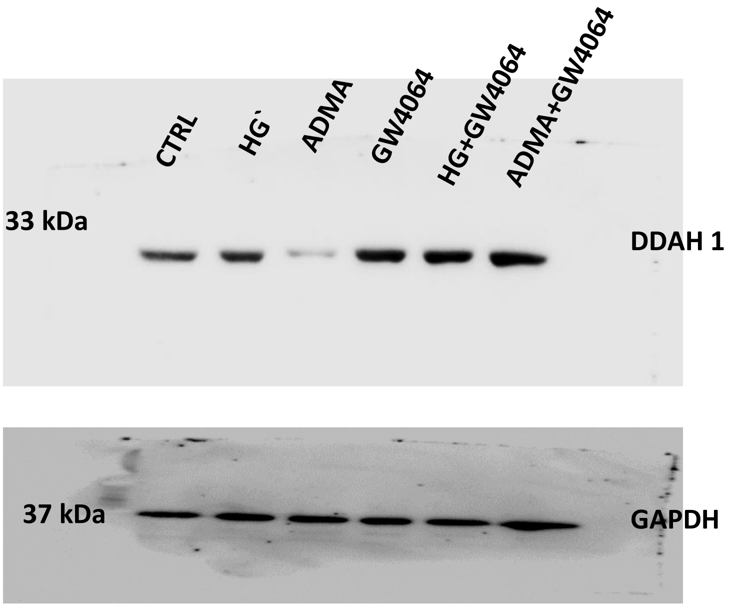

Supplement: Supplementary file 1 — Supplementary Information [file 41598_2020_72943_MOESM1_ESM.pdf]
